# Deep Learning applications for COVID-19

**DOI:** 10.1186/s40537-020-00392-9

**Published:** 2021-01-11

**Authors:** Connor Shorten, Taghi M. Khoshgoftaar, Borko Furht

**Affiliations:** Florida Atlantic University, 777 Glades Road, Boca Raton, FL 33431 USA

**Keywords:** COVID-19, Deep Learning applications, Natural Language Processing, Computer Vision, Life Sciences, Epidemiology

## Abstract

This survey explores how Deep Learning has battled the COVID-19 pandemic and provides directions for future research on COVID-19. We cover Deep Learning applications in Natural Language Processing, Computer Vision, Life Sciences, and Epidemiology. We describe how each of these applications vary with the availability of big data and how learning tasks are constructed. We begin by evaluating the current state of Deep Learning and conclude with key limitations of Deep Learning for COVID-19 applications. These limitations include Interpretability, Generalization Metrics, Learning from Limited Labeled Data, and Data Privacy. Natural Language Processing applications include mining COVID-19 research for Information Retrieval and Question Answering, as well as Misinformation Detection, and Public Sentiment Analysis. Computer Vision applications cover Medical Image Analysis, Ambient Intelligence, and Vision-based Robotics. Within Life Sciences, our survey looks at how Deep Learning can be applied to Precision Diagnostics, Protein Structure Prediction, and Drug Repurposing. Deep Learning has additionally been utilized in Spread Forecasting for Epidemiology. Our literature review has found many examples of Deep Learning systems to fight COVID-19. We hope that this survey will help accelerate the use of Deep Learning for COVID-19 research.

## Introduction

SARS-CoV-2 and the resulting COVID-19 disease is one of the biggest challenges of the 21st century. At the time of this publication, about 43 million people have tested positive and 1.2 million people have died as a result [1]. Fighting this virus requires heroism of healthcare workers, social organization and technological solutions. This survey focuses on advancing technological solutions, with an emphasis on Deep Learning. We additionally highlight many cases where Deep Learning can facilitate social organization such as Spread Forecasting, Misinformation Detection, or Public Sentiment Analysis. Deep Learning has gained massive attention by defeating the world champion at Go [2], controlling a robotic hand to solve a Rubik’s cube [3], and completing fill-in-the-blank text prompts [4]. Deep Learning is advancing very quickly, but what is the current state of this technology? What problems does Deep Learning have the capability of solving? How do we articulate COVID-19 problems for the application of Deep Learning? We explore these questions through the lens of Deep Learning applications fighting COVID-19 in many ways.

This survey aims to illustrate the use of Deep Learning in COVID-19 research. Our contributions are also follows:

- This is the first survey viewing COVID-19 applications solely through the lens of Deep Learning. In comparison with other surveys on COVID-19 applications in Data Science or Machine Learning, we provide extensive background on Deep Learning.
- For each application area surveyed, we provide a detailed analysis of how the given data is inputted to a deep neural network and how learning tasks are constructed.
- We provide an exhaustive list of applications in data domains such as Natural Language Processing, Computer Vision, Life Sciences, and Epidemiology. We particularly focus on work in Literature Mining for COVID-19 research papers, compiling papers from the ACL 2020 NLP-COVID workshop.
- Finally, we review common limitations of Deep Learning including Interpretability, Generalization Metrics, Learning from Limited Labeled Data, and Data Privacy. We describe how these limitations impact each of the surveyed COVID-19 applications. We additionally highlight research tackling these issues.

Our survey is organized into four primary sections. We start with a “Background” on Deep Learning to explain the relationship with other Artificial Intelligence technologies such as Machine Learning or Expert Systems. This background also provides a quick overview of SARS-CoV-2 and COVID-19. The next section lists and explains “Deep Learning applications for COVID-19”. We organize surveyed applications by input data type, such as text or images. This is different from other surveys on COVID-19 that organize applications by scales such as molecular, clinical, and society-level [5, 6].

From a Deep Learning perspective, organizing applications by input data type will help readers understand common frameworks for research. Firstly, this avoids repeatedly describing how language or images are inputted to a Deep Neural Network. Secondly, applications working with the same type of input data have many similarities. For example, cutting-edge approaches to Biomedical Literature Mining and Misinformation Detection both work with text data. They have many commonalities such as the use of Transformer neural network models and reliance on a self-supervised representation learning scheme known as language modeling. We thus divide surveyed COVID-19 applications into “Natural Language Processing”, “Computer Vision”, “Life Sciences”, and “Epidemiology”. However, our coverage of applications in Life Sciences diverges from this structure. In the scope of Life Sciences, we describe a range of input data types, such as tabular Electronic Health Records (EHR), textual clinical notes, microscopic images, categorical amino acid sequences, and graph-structured network medicine.

The datasets used across these applications tend to share the common limitation of size. In a rapid pandemic response situation, it is especially challenging to construct large datasets for Medical Image Analysis or Spread Forecasting. This problem is evident in Literature Mining applications such as Question Answering or Misinformation Detection as well. Literature Mining data is an interesting situation for Deep Learning because we have an enormous volume of published papers. Despite having such a large unlabeled dataset, downstream applications such as question answering or fact verification datasets are extremely small in comparison. We will continually discuss the importance of pre-training for Deep Learning. This paradigm relies on either supervised or self-supervised transfer learning. Of core importance, explored throughout this paper, is the presence of in-domain data. Even if it is unlabeled, such as a biomedical literature corpus, or slightly out-of-domain, such as the CheXpert radiograph dataset for Medical Image Analysis [7], availability of this kind of data is paramount for achieving high performance.

When detailing each application of Deep Learning to COVID-19, we place an emphasis on the representation of data and the task. Task descriptions mostly describe how a COVID-19 application is constructed as a learning problem. We are solely focused on Deep Learning applications, and thus we are referring to representation learning of raw, or high-dimensional data. A definition and overview of representation learning is provided in “Background” section. The following list quickly describes different learning variants found in our surveyed applications:

- Supervised Learning optimizes a loss function with respect to predicted and ground truth labels. These ground truth labels require manual annotation.
- Unsupervised Learning does not use labels. This includes clustering algorithms that look for intrinsic structure in data.
- Self-Supervised Learning optimizes a loss function with respect to the predicted and ground truth labels. Differently from Supervised Learning, these labels are constructed from a separate computing process, rather than human annotation.
- Semi-Supervised Learning uses a mix of human labeled and unlabeled data for representation learning.
- Transfer Learning describes initializing training with the representation learned from a previous task. This previous task is most commonly ImageNet-based supervised learning in “Natural Language Processing” or Internet-scale language modeling in “Computer Vision”.
- Multi-Task Learning simultaneously optimizes multiple loss function, usually either interleaving updates or applying regularization penalties to avoid conflicting gradients from each loss.
- Weakly Supervised Learning refers to supervised learning with heuristically labeled data, rather than carefully labeled data.
- Multi-Modal Learning describes representation learning in multiple data types simultaneously, such as images and text or images and electronic health records.
- Reinforcement Learning optimizes a loss function with respect to a series of state to action predictions. This is especially challenging due to credit assignment in the sequence of state to action mappings when receiving sparse rewards.

It is important to note the distinction between these learning task constructions in each of our surveyed applications. We further contextualize our surveyed applications with an overview of “Limitations of Deep Learning”. These limitations are non-trivial and present significant barriers for Deep Learning to fight COVID-19 related problems. Solutions to these issues of “Interpretability”, “Generalization metrics”, “Learning from limited labeled datasets”, and “Data privacy” will be important to many applications of Deep Learning. We hope describing how the surveyed COVID-19 applications are limited by these issues will develop intuition about the problems and motivate solutions. Finally, we conclude with a “Discussion” and “Conclusion” from our literature review. Our Discussion describes lessons learned from a comprehensive literature review and plans for future research.

Deep Learning for “Natural Language Processing” (NLP) has been extremely successful. Applications for COVID-19 include Literature Mining, Misinformation Detection, and Public Sentiment Analysis. Searching through the biomedical literature has been extremely important for drug repurposing. A success case of this is the repurposing of baricitinib [8], an anti-inflammatory drug used in rheumatoid arthritis. The potential efficacy of this drug was discovered by querying biomedical knowledge graphs. Modern knowledge graphs utilize Deep Learning for automated construction. Other biomedical literature search systems use Deep Learning for information retrieval from natural language queries. These Literature Mining systems have been extended with question answering and summarization models that may revolutionize search altogether. We additionally explore how NLP can fight the “infodemic” by detecting false claims and presenting evidence. NLP is also useful to evaluate public sentiment about the pandemic from data such as tweets and provide tools for social scientists to analyze free-text response surveys.

“Computer Vision” is another mature application domain of Deep Learning. The Transformer revolution in Natural Language Processing largely owes its success to Computer Vision’s pioneering into large datasets, massive models, and the utilization of hardware that accelerates parallel computation, namely GPUs [9]. Computer Vision applications to COVID-19 include Medical Image Analysis, Ambient Intelligence, and Vision-based Robotics. Medical Image Analysis has been used to supplement RT-PCR testing for diagnosis by classifying COVID-induced pneumonia from chest X-rays and CT scans. Haque et al. [10] recently published a survey on Computer Vision applications for physical space monitoring in hospitals and daily living spaces. They termed these applications “Ambient Intelligence”. This is an interesting phrase to encompass a massive set of more subtle applications such as automated physical therapy assistance, hand washing detection, or surgery training and performance evaluation. This section is particularly suited to our discussion on Data Privacy in “Limitations of Deep Learning”. We also look at how Vision-Based Robotics can ease the economic burden of COVID-19, as well as automate disinfection.

Deep Learning can improve virus spread models used in “Epidemiology”. Our coverage of these models starts with “black-box” forecasting. These models use a history of infections, as well as information such as lockdown phase, to predict future cases or deaths. We describe how this varies based on region specificity. We will then look at adding more structure to the population model. The most well-known example of this are Susceptible, Infected, and Recovered (SIR) models. The illustrative SIR model describes how a population transitions from healthy or “Susceptible”, to “Infected”, and “Recovered” through a set of three differential equations. These equations solve for the infection and recovery rates from data of initial and recovered populations. The challenge with these SIR models is that they have limiting assumptions. We will explore how Deep Neural Networks have been used to solve differential equations and integrate the non-linear impact of quarantine or travel into these SIR models. For even finer-grained predictions, we looked into the use of Contact Tracing, potentially enabling personalized risk of infection analysis.

The application of Deep Learning for “Life Sciences” is incredibly exciting, but still in its early stages. RT-PCR has become the gold standard for COVID-19 testing. This viral nucleic acid test utilizes primers and transcription enzymes to amplify a chunk of DNA such that fluorescent probes can signal the presence of the viral RNA. However, these tests have a high false negative rate. We will look at studies that sequence this RNA and deploy deep classification models, use Computer Vision to process expression, as well as studies that design assays to cover a wide range of genomes. New diagnostic tools are being developed with detailed biological and historical information about each patient. This is known as Precision Medicine. Precision Medicine in COVID-19 applications looks at predicting patient outcome based on patient history recorded in Electronic Health Records (EHR), as well as miscellaneous biomarkers such as blood testing results. This is another section that is highly relevant for our cautionary “Limitations of Deep Learning” with respect to Data Privacy.

Another exciting application area is the intersection of Deep Learning and molecular engineering. Deep Learning has received massive press for the development of AlphaFold. Given the 1-dimensional string of amino acids, AlphaFold predicts the resulting 3-D structure. These models have been used to predict the 3-D structure of the spike proteins on the outer shell of the coronavirus, as well as its other proteins. Having a model of this structure allows biochemists to see potential binding targets for drug development. These bindings can prevent the virus from entering human cells through membrane proteins such as ACE2. We can use Deep Learning to suggest potential binding drug candidates. Developing new drugs will have to undergo a timely and costly clinical trial process. For this reason, COVID-19 research has been much more focused on drug repurposing to find treatments.

Within the scope of Natural Language Processing, we present the automated construction of biomedical knowledge graphs from a massive and rapidly growing body of literature. These graphs can be used to discover potential treatments from already approved drugs, an application known as drug repurposing. Drug repurposing is highly desirable because the safety profile of these drugs has been verified through a rigorous clinical trial process. Biomedical experts can search through these knowledge graphs to find candidate drugs. However, another interesting way to search through these graphs is to set up the problem as link prediction. Given a massive graph of nodes such as proteins, diseases, genes and edges such as “A inhibits B”, we can use graph representation learning techniques such as graph neural networks to predict relations between nodes in the graph.

Our final application area surveyed is the use of Deep Learning for “Epidemiology”. How many people do we expect to be infected with COVID-19? How long do we have to quarantine for? These are query examples for our search systems, described as NLP applications, but epidemiological models are the source of these answers. We will begin exploring this through the lens of “black-box” forecasting models that look at the history of infections and other information to predict into the future. We will then look at SIR models, a set of differential equations modeling the transition from Susceptible to Infected to Recovered. These models find the reproductive rate of the virus, which can characterize the danger of letting herd immunity develop naturally. To formulate this as a Deep Learning task, a Deep Neural Network approximates the time-varying strength of quarantine in the SIR model, since integrating the Exposed population would require extremely detailed data. Parameter optimizers from Deep Learning such as Adam [11] can be used to solve differential equations as well. We briefly investigate the potential of Contact Tracing data. Tracking the movement of individuals when they leave their quarantine could produce incredibly detailed datasets about how the virus spreads. We will explore what this data might look like and some tasks for Deep Learning. This is another application area that relates heavily to our discussion of data privacy in “Limitations of Deep Learning”.

These applications of Deep Learning to fight COVID-19 are promising, but it is important to be cognizant of the drawbacks to Deep Learning. We focus on the issues of “Interpretability”, “Generalization metrics”, “Learning from limited labeled datasets”, and “Data Privacy”. It is very hard to interpret the output of current Deep Learning models. This problem is further compounded by the lack of a reliable measure of uncertainty. When the model starts to see out-of-distribution examples, data points sampled from a different distribution than the data used to train the model, most models will continue to confidently misclassify these examples. It is very hard to categorize how well a trained model will generalize to new data distributions. Furthermore, these models can fail at simple commonsense tasks, even after achieving high performance on the training dataset. Achieving this high performance in the first place comes at the cost of massive, labeled datasets. This is unpractical for most clinical applications like Medical Image Analysis, as well as for quickly building question-answering datasets. Finally, we have to consider data privacy with these applications. Will patients feel comfortable allowing their ICU activity to be monitored by an intelligent camera? Would patients be comfortable with their biological data and medical images being stored in a central database for training Deep Learning models? This introduction should moderate enthusiasm about Deep Learning as a panacea to all problems. However, we take an optimistic look at these problems in our section “Limitations of Deep Learning”, explaining solutions to these problems as well, such as self-explanatory models or federated learning.

## Background

This section will provide a background for this survey. We begin with a quick introduction to COVID-19, followed by what Deep Learning is and how it relates to other Artificial Intelligence technologies. Finally, we present the relationship of this survey with other works reviewing the use of Artificial Intelligence, Data Science, or Machine Learning to fight COVID-19.

SARS-CoV-2 originated from Wuhan, China and spread across the world, causing a global pandemic. The response has been a mixed bag of mostly chaos and a little optimism. Scientists were quick to sequence and publish the complete genome of the virus [12], and individuals across the world quarantined themselves to contain the spread. Scientists have lowered barriers for collaboration. However, there have been many negative issues surrounding the pandemic. The quick infection and lack of resources has overloaded hospitals and heavily burdened healthcare workers. SARS-CoV-2 has a unique characteristic of peak infection before symptom manifestation that has worked in the favor of the virus. Misinformation has spread so rampantly, a new field of “infodemiology” has sprouted to fight the “infodemic”. Confusion of correct information is compounded by a rapidly growing body of literature surrounding SARS-CoV-2 and COVID-19. This research is emerging very quickly and new tools are needed to help scientists organize this information.

A technical definition of Deep Learning is the use of neural networks with more than one or two layers. A neural network “layer” is typically composed of a parametric, non-linear transformation of an input. Stacking these transformations forms a statistical data structure capable of mapping high-dimensional inputs to outputs. This mapping is executed by optimizing the parameters. Gradient descent is the tool of choice for this optimization. Gradient descent works by taking the partial derivative of the loss function with respect to each of the parameters, and updating the parameters to minimize the loss function. Deep Learning gets the name “Deep” in reference to stacking several of these layers. The second part of the name, “Learning”, references parameter optimization. State-of-the-art Deep Learning models typically contain between 100 million to 10 billion parameters and 50–200 layers. Two of the largest models publicly reported are composed of 600 and 175 billion parameters [4, 13]. Scaling up the size of these models has accelerated extremely quickly in the past few years [14].

In application of data such as text, images, or molecular sequences, Deep Learning is a massive step up from Machine Learning. This is because Deep Learning learns these features automatically, compared to Machine Learning where features are manually-constructed. Machine Learning also describes fitting parametric models to map from input to output. Machine Learning processes inputs represented by human crafted features. A crafted feature to classify an animal as a dog or a zebra could be the weight of the animal, or the possession of stripes.

For high-dimensional data such as image pixel tensors or text embedding matrices, it is very hard to manually design high performing features for Machine Learning. Machine Learning is much different from Deep Learning, in which the features are learned automatically from this high-dimensional “raw” data. The features learned in Deep Learning are referred to as representations. A representation is typically analyzed through the penultimate vector that is inputted to the output prediction. It is very challenging to interpret this representation because of the non-linear interactions between variables that lead to it. For example, we cannot say the 3rd and 8th position of the representation vector solely look for the possession of stripes. In addition to looking at the penultimate layer vector output, the representation is also examined through the first embedding vector for word tokens. In either case, these high-dimensional vectors are commonly visualized through dimensionality reduction techniques such as t-SNE [15] or UMAP [16].

The entire set of intermediate neural network outputs can equally be considered as the representation of the data. It is very important to view representations in this way for the sake of transfer learning. This is where a neural network is trained on one task, usually one with a much larger set of labeled data, and then sequentially trained on another task. The difference between human-designed features and representation learned from raw data is the core distinction between Deep and Machine Learning.

Deep Learning is a piece in the bigger picture of Artificial Intelligence (AI). In addition to the distinction between Deep and Machine Learning, the scope of AI also includes Symbolic Systems. Symbolic Systems produce intelligent behavior through symbol manipulation and logic. Examples include Expert Systems and Knowledge Graphs. Expert Systems uses if-else rules to make decisions. Knowledge Graphs store relations between objects in graph data structures. The application of Knowledge Graphs is extremely useful for fighting COVID-19, an example of this is BenevolentAI’s Knowledge Graph [17]. This is done by searching through explicitly coded relations between proteins, drugs, and clinical trial observations, to name a few. Biomedical researchers use a structured query language, rather than natural language, to search through these graphs.

Deep Learning does not process information in the same way as Symbolic Systems. Rather than topological compositions of atomic units, Deep Learning stores information in distributed tensors. There is an interplay with Deep Learning in symbolic systems like Knowledge Graphs. Deep Learning is used to automate the construction of Knowledge Graphs through Named Entity Recognition and Relation Extraction tasks. Manually performing these tasks on big datasets such as a corpus of biomedical literature would be impossible. This automated Knowledge Graph construction is discussed heavily in our survey in application to drug repurposing.

We recommend readers explore Chollet’s Measure of Intelligence [18] for a definition of intelligence more generally. Intelligence is defined as a function of prior knowledge, experience, and generalization difficulty. This is a useful framework for thinking about the intelligence required with surveyed COVID-19 applications. What makes one application require more intelligence than another? How can we add more prior knowledge to these systems? How might this prior knowledge limit generalization ability? It is argued that we can trade off more prior knowledge for less experience, or vice-versa, we can start with less prior knowledge and make up for that with more experience. The success of these components are determined by the generalization difficulty of the task. Different kinds of prior knowledge injected into an artificial intelligence may limit generalization ability, as will different subsets of experience. The efforts of Deep Learning research can be thought of as discovering mechanisms of prior knowledge, collecting experience, and measuring generalization difficulty.

The current generation of Deep Learning is defined in our survey as sequential processing networks with many layers, updating its parameters with a global loss function, and forming distributed representations of data. We have seen an evolution from Machine Learning in representation learning. We also seek to integrate Symbolic Systems, such as the use of Knowledge Graphs. We think it is useful for readers to think of the interplay between prior knowledge, experience, and generalization difficulty to frame the difficulty of our surveyed applications.

**Fig. 1:**
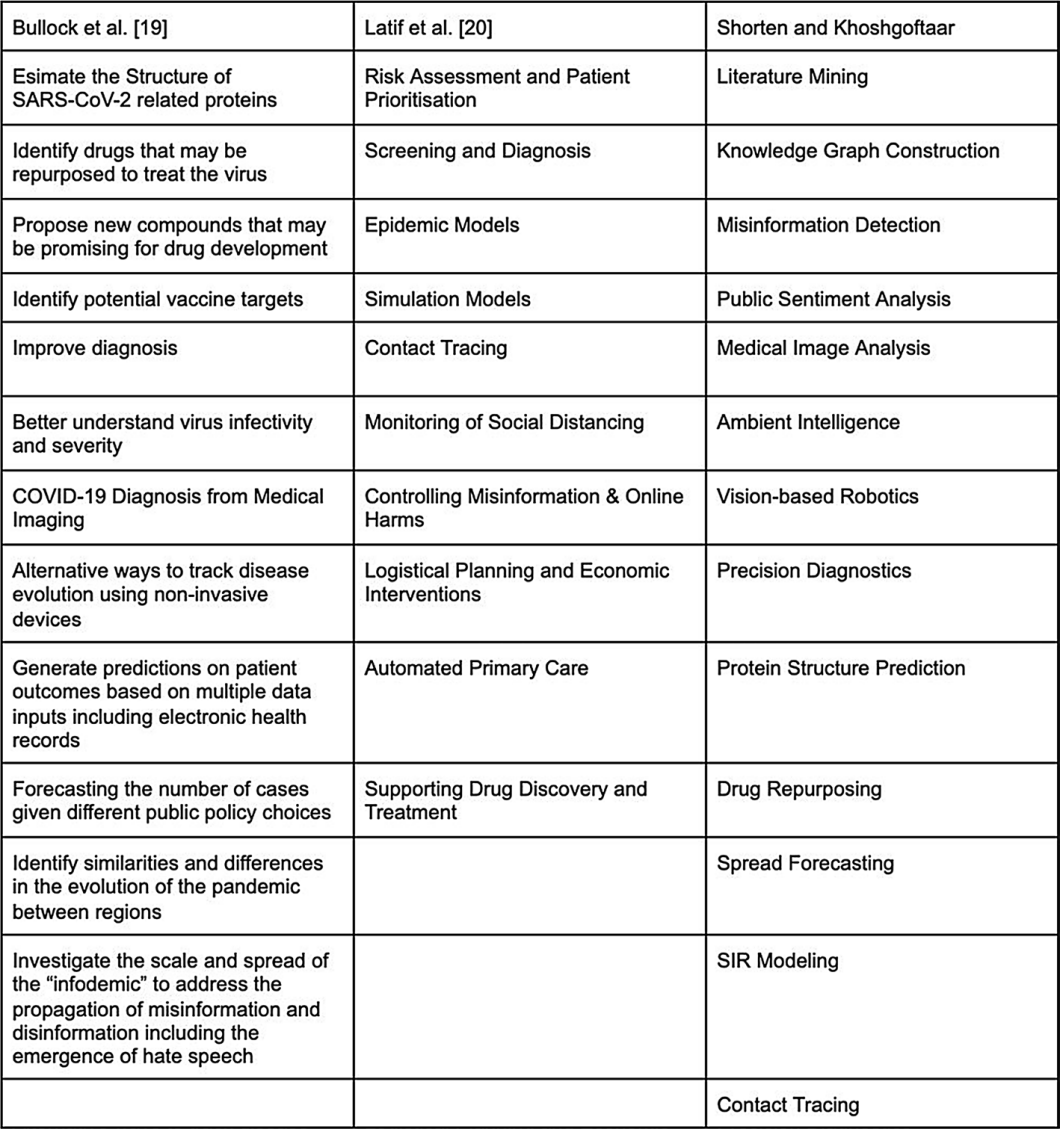
Organization of Artificial Intelligence COVID-19 Applications, comparison with other literature surveys

Many other researchers have surveyed the use of Artificial Intelligence to fight COVID-19. Our survey builds on these reports, with a more detailed dive into Deep Learning. Surveys covering AI, Data Science, or Machine Learning applied to COVID-19 vary mostly in how they organize COVID-19 applications. For example, Bullock et al. [5] organize their survey into molecular, clinical, and societal perspectives. Figure 1 illustrates how we have deviated from other surveys in presenting COVID-19 applications. Most notably, we do not cover the use of Deep Learning for audio data or the Internet of Things (IoT). Furthermore, applications we do not cover include the diagnostic potential of audio data from breathing recordings [6], and IoT applications such as smartphone temperature and inertial sensors [19]. Contrary to other surveys, we integrate publicly available datasets into our applications, rather than separate the two topics.

Bullock et al. [5] describe the aim of their survey as “not to evaluate the impact of the described techniques, nor to recommend their use, but to show the reader the extent of existing applications and to provide an initial picture and road map of how Artificial Intelligence could help the global response to the COVID-19 pandemic”. We have a similar aim in our survey, focusing solely on Deep Learning. Our survey draws heavy inspiration from Raghu and Schmidt’s paper, “A Survey of Deep Learning for Scientific Discovery” [20]. They cover different Deep Learning models, variants to the supervised learning training process, and limitations of Deep Learning, most notably reliance on large, labeled datasets. Our survey aims to provide a similar overview of Deep Learning and how it can be adapted to different kinds of scientific problems, focused on COVID-19.

## Deep Learning applications for COVID-19

### Natural Language Processing

We begin our coverage of Natural Language Processing (NLP) by describing how text data is inputted to Deep Neural Networks. In order to feed language as an input to a Deep Neural Network, words are first tokenized into smaller components and mapped to an index in an embedding table. We take a token such as “cat” and map it into a d-dimensional embedding vector, where d is described as the hidden dimension of the Deep Neural Network. For further illustration, the token “the” might be mapped to position “810” in an index table the size of the entire vocabulary. Each of these positions holds a d-dimensional embedding vector representing a unique token. Note this strategy can be used for any categorical variable input. This input representation has been very successful with language tokens. This strategy is used for other categorical variable encodings as well, such as amino acids tokens.

NLP has seen a boom of interest due to the invention of the Transformer Neural Network architecture [21]. This marks a transition from a focus on Recurrent Neural Networks (RNNs). RNNs iteratively process a sequence piece by piece, usually with explicit internal memory such as the Long Short-Term Memory (LSTM) models. The main attraction of the Transformer is the use of attention layers. The attention layer was invented to help RNNs preserve information from early tokens in the sequence. The famous paper “Attention is all you Need” [21], showed that attention layers are potent enough on their own to do away with recurrent sequence processing. Another benefit of this is the ability to massively parallelize the computation in the networks. The importance of this parallelization is best described with a quick history of AlexNet in Computer Vision.

The success of AlexNet [22] in the Computer Vision task of image classification was a large driver of interest in Deep Learning. AlexNet is an implementation of a Convolutional Neural Network, a new architecture at the time that has since been widely adopted. The forward and backward computation in Convolutional and Transformer Neural Networks can run in parallel. Parallelization enables massive computing acceleration from Graphics Processing Units (GPUs). A similar breakthrough has happened in NLP with Transformers. This perfect marriage with parallel GPU computing has dramatically improved Deep Learning performance.

Scaling up Transformers allows them to take advantage of big data, a necessary component of Deep Learning success described further in “Limitations of Deep Learning”. Another reason for the advancement of NLP is the success of self-supervised pre-training and transfer learning. It would be extremely challenging to find a big dataset of question-answer pairs related to COVID-19. However, we can find big data in the entire corpus of research published on SARS-CoV-2 and COVID-19. This data is not labeled. We cannot rely on supervised learning to learn representations from this data. The solution to this has been self-supervised language modeling. Language models mask out a token randomly and the model predicts what the masked token had originally been. The term “self-supervised” comes from the way this task can use supervised loss functions such as cross-entropy loss on the predicted token, but the task is constructed without human annotation.

After self-supervised language modeling on a large corpus, the model is transferred to a new task, such as Yelp review sentiment classification. The initialization of the neural network from the weights learned by language modeling is an incredibly powerful starting point. Gururang et al. [23] show the importance of in-domain data for this self-supervised pre-training. General-purpose language models such as BERT [24] or GPT [25] are trained on a massive corpus, such as all the text on Wikipedia, a massive set of books, and articles sourced from the internet. Language models repurposed for COVID-19 literature mining tasks such as BioBERT [26] or SciBERT [27] are pre-trained on a more domain-relevant corpus of scientific papers and biomedical literature. Another example, COVID-BERT [28] is pre-trained on a corpus of tweets about COVID-19. In-domain pre-training is extremely important for the success of transfer learning for COVID-19 NLP tasks. We will return to this theme in our discussion of Medical Image Analysis as well.

**Fig. 2:**
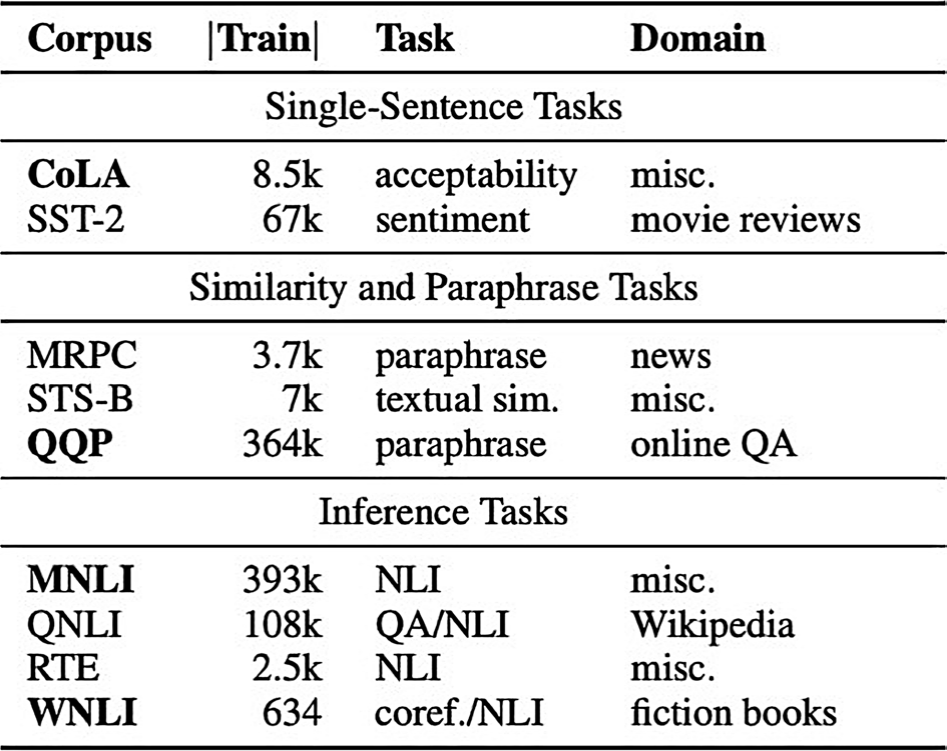
What tasks has NLP conquered? A quick overview of the number of examples, tasks, and domains contained in the GLUE benchmark (Image taken from Wang et al. [29])

We present NLP applications for COVID-19 ordered by difficulty with respect to current Deep Learning systems. Figure 2 is a quick description of the GLUE NLP benchmark. This table gives information about each task such as how many training examples are in each dataset, a quick description of the task, and a high-level summary of the data domain [29]. The GLUE benchmark is a set of tasks to evaluate NLP systems. The latest NLP systems perform so well at these tasks that a new benchmark, SuperGLUE [30] has since been designed. Starting with the GLUE benchmark should provide readers a solid foundation for understanding what current NLP can easily solve. We explain how these tasks are setup as a Deep Learning problem to help readers understand the similarities of GLUE tasks with our surveyed COVID-19 applications. We will then transition to adapting these task formulations to COVID-19 applications.

The GLUE benchmark divides supervised learning tasks into categories of Single-Sentence, Similarity and Paraphrase, and Inference. These categories mostly distinguish the input format for classification tasks. Single-Sentence deals with one sentence as input, whereas similarity and inference deal with two. On the GLUE benchmark, this text is sentence-length sequences. The length of the sequence is an important distinction to make. Intuitively, it might seem easier to classify a longer sequence, such as an entire COVID-19 clinical report; however, attention over a long document has a much higher computational cost than sentence-length input sequences.

In the GLUE benchmark, single sentences are classified based on if they are grammatically acceptable or if they are positive or negative in sentiment. Text Classification applications in COVID-19 include certain approaches to Misinformation Detection, Public Sentiment Analysis, topic classification, and question category classification. Misinformation Detection and Public Sentiment Analysis research usually work with Twitter data. Tweets are great for NLP models since they are limited to 280 characters. An example of this is COVID-Twitter-BERT from Muller et al. [28].

Topic classification with scientific papers addressing COVID-19 requires constructing a heavily truncated atomic unit for papers. We cannot pass entire scientific papers as input to most NLP models. An application example of this is filtering out COVID-19 papers focused solely on Radiological findings from Liang and Xie [31]. Another example of this task is COVID-19 question classification from Wei et al. [32]. Wei et al. fine-tune BERT to categorize public questions about COVID-19 into categories such as transmission, societal effects, prevention, and more. This helps public officials understand what the public is concerned about with respect to COVID-19.

Similarity tasks in the GLUE benchmark are based on telling if two text sequences have the same semantic meaning. In GLUE, this is explored on miscellaneous data sources, news snippets, and questions asked on the Quora social network. In applications to Misinformation Detection, we might have a list of common rumors about COVID-19. We can use these semantic similarity models to detect when these rumors are being spread on social media. This detection is much more flexible than keyword models that would simply look for terms like “5G”. Semantic similarity models also play an enormous role in information retrieval systems.

Information retrieval models try to find the most relevant information given a query. This is executed by performing the same task, but at multiple stages of granularity. Generally, we will consider these stages of high recall and high precision as retrieval and re-ranking. Retrieval and re-ranking could be further decomposed to trade-off between higher precision and more computation. The first retrieval stage has typically been done by hand-crafted TF-IDF and BM25 text features. Only very recently has information retrieval looked at transformer-derived representations for the first retrieval stage [33]. The second stage of re-ranking takes the retrieved documents as input and assigns a more precise relevant score for each document, sorting them by the highest score to answer the query.

The next task in NLP relevant to COVID-19 applications is question answering (QA). QA has been well studied on the SQuAD benchmark [34]. This formulation of question answering (QA) is referred to as extractive QA. The model has to classify the answer as a start and end span within a provided chunk of context. The supervised learning problem is to output the indices from the context. For COVID-19 extractive question answering, an important question is, how do we get the context to classify the answer in? One solution would be to pair extractive QA with the information retrieval systems previously described to provide context to find the answer span. In addition to SQuAD-style questions, we look at complex question answering. Complex, or multi-hop, question answering requires the model to combine information from different sources to derive the answer.

Some approaches to QA surveyed generate the answer directly [35], rather than classifying its presence in a context. We consider this formulation to be more akin to abstractive question answering. We will explore two approaches to QA, either using symbolic knowledge graphs or relying on a neural system. A symbolic knowledge graph is desirable for the sake of interpretability. However, manual relation labeling in the COVID-19 literature does not scale. Neural systems still play a role in constructing these knowledge graphs through Named Entity Recognition and Relation Extraction tasks. The alternative approach to using symbolic knowledge graphs is to rely solely on Deep Learning models. In this case, a Deep Learning model stores all the information it needs to answer questions in its parameters [36]. We refer readers to [37] for current work on Knowledge Intensive Tasks in NLP.

Another NLP task that is interesting for COVID-19 applications is abstractive question answering, summarization, and chatbots. These tasks require the model to devise novel sequences of text to answer questions, summarize articles, or chat with a user. We view each of these application areas as facing the same problem, differing with respect to the size of the input and output. All of these applications may be expected to contain a massive amount of information, whether that information is accessed directly in the parameters of the model or in the input context window. We note that these types of models are still in the early stage of development. We survey Abstractive QA and summarization models implemented in Literature Mining systems such as CO-Search [38] and CAiRE-COVID [39].

The ambition of NLP tasks in COVID-19 research range on the scale from well studied GLUE-style problems to extractive question answering when the context is provided, and then up to chatbots and abstractive summarization. We note that at the time of this publication, reliable abstractive summarization is a moonshot application of Deep Learning. Artificial Intelligence is an exciting and imagination-provoking technology. The results from GPT-3, a 175 billion parameter language model, has been extremely inspiring. COVID-19 researchers are certainly pushing the limits of what NLP can do.

#### Literature Mining

The COVID-19 pandemic ignited a call to arms of scientists across the world. Consequentially, searching for signal in the noise is more challenging. The most popular open literature dataset, CORD-19 [40], contains over 128,000 papers. These papers contain information about SARS-CoV-2, as well as related coronaviruses such as SARS and MERS, information about COVID-19, and relevant papers in relation to drug repurposing. No single or group of human beings could be expected to read this amount of text. The need to organize a massive scale of text data has inspired development of many NLP systems.

CORD-19: The COVID-19 Open Research Dataset [40] is the most popular dataset containing this growing body of literature. The dataset consists of data from publications and preprints on COVID-19, as well as historical coronaviruses such as SARS and MERS. These papers are sourced from PubMed Central (PMC), PubMed, the World Health Organization’s COVID-19 database, and preprint servers such as bioRxiv, medRxiv, and arXiv. The CORD-19 research paper documents the rapid growth of their dataset from an initial release of 28,000 papers when published on April 22nd, up to 140,000 when revised on July 10th. We observed documentation of this explosive growth as well when surveying Literature Mining systems built on top of this dataset. The CORD-19 data is cleaned and implemented with the same system used for the Semantic Scholar Open Research Corpus [41].

**Fig. 3:**
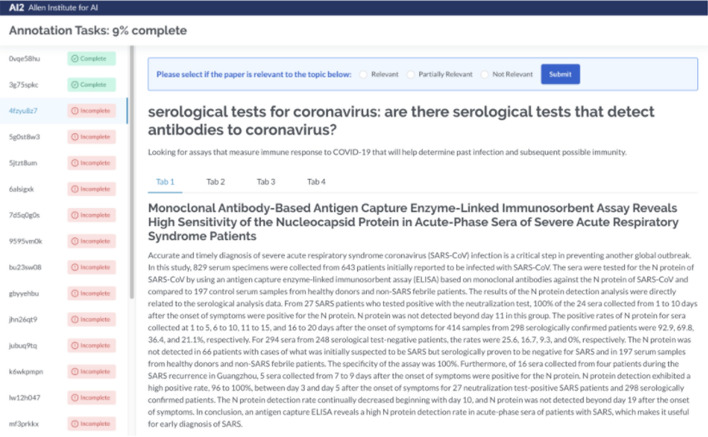
Interface for human labeling TREC-COVID documents (Image taken from Voorhees et al. [43])

Whereas CORD-19 is general purpose, TREC-COVID [42] is more narrowly focused on a test evaluation of information retrieval. The authors state the twin goals of the dataset are “to evaluate search algorithms and systems for helping scientists, clinicians, policy makers, and others manage the existing and rapidly growing corpus of scientific literature related to COVID-19 and to discover methods that will assist with managing scientific information in future global biomedical crises” [43]. The TREC-COVID dataset consists of topics where each topic is composed of a query, question, and narrative. The narrative is a longer description of the question. Figure 3 shows an example of the interface the authors use to label the TREC-COVID dataset.

The following list provides a quick description of some Literature Mining systems built from datasets such as CORD-19 and TREC-COVID. These systems use a combination of Information Retrieval, Knowledge Graph Construction, Question Answering, and Summarization to facilitate exploration into the COVID-19 scientific literature.

**Fig. 4:**
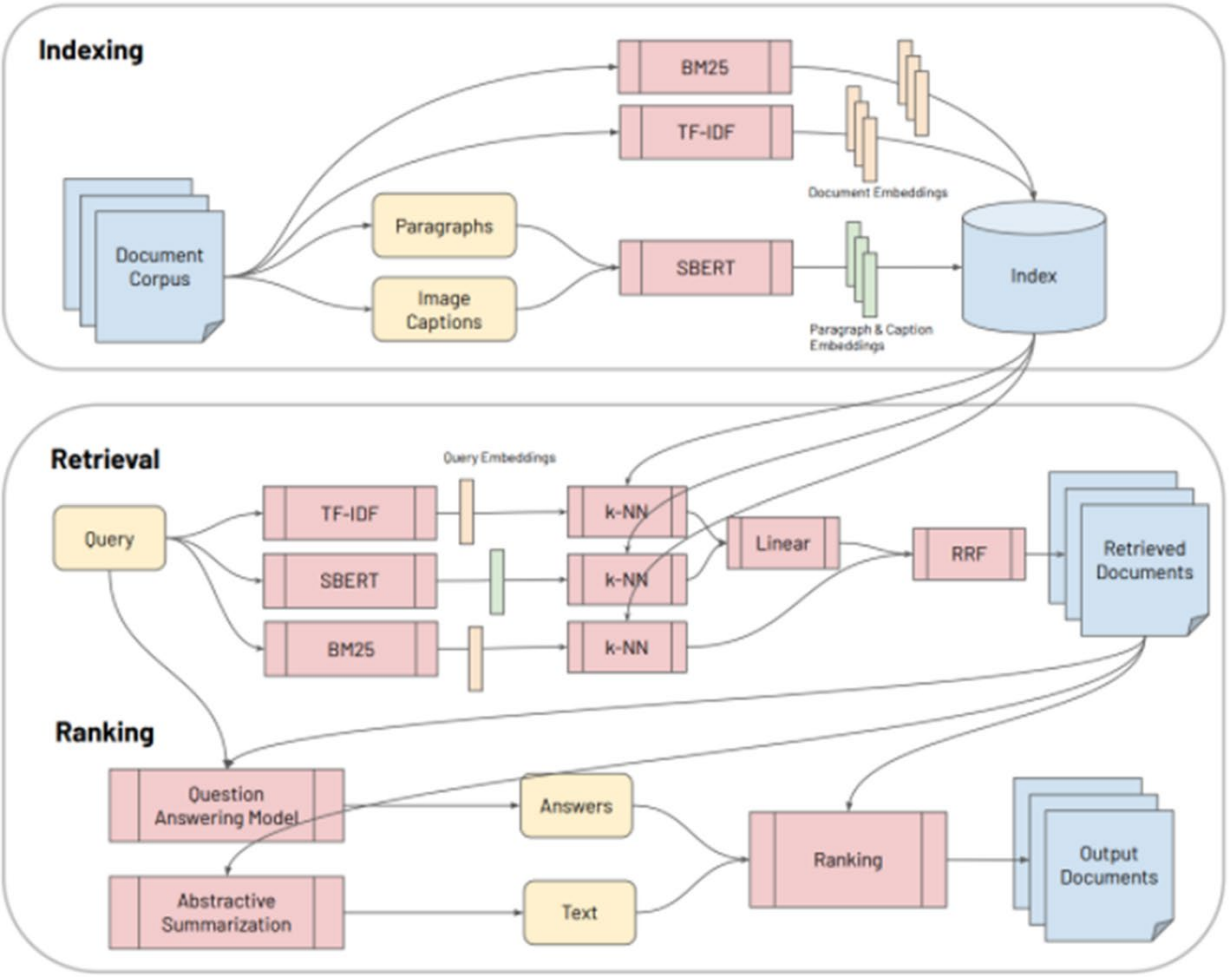
CO-Search System Architecture (Image taken from Esteva et al. [38])
- Covidex [45] is a Retrieve-then-Rank system combining keyword retrieval with neural re-ranking. The most different aspects of Covidex as compared to CO-Search are a sequence-to-sequence (seq2seq) approach to re-ranking and bootstraps the training of this model from the MS MARCO [46] passage ranking dataset. The MS MARCO dataset contains 8.8M passages obtained from the top 10 results by the Bing search engine, corresponding to 1M unique queries. The monoT5 [47] seq2seq re-ranker takes as input “Query q: Document: d Relevant: “ to classify whether the document is relevant or not to the query.
- SLEDGE [48] deploys a similar pipeline of keyword-based retrieval followed by neural ranking. Differently from Covidex, SLEDGE utilizes ths SciBERT [27] model for re-ranking. SciBERT is an extension to BERT that has been pre-trained on Scientific Text. Additionally, the authors of SLEDGE find large gains by integrating the publication date of the articles into the input representation.
- CAiRE-COVID [39] is a similar system to CO-Search. The primary difference comes from the use of the MRQA [49] model and avoiding fine-tuning the QA models on COVID-19 related datasets. This system tests the generalization of existing QA models comprising of a pre-trained BioBERT [26] fine-tuned on the SQuAD dataset. The user interface of CAiRE-COVID is depicted in Fig. 5

- CO-Search [38] is a Retrieve-then-Rank system composed of many parts, shown in Fig. 4. Before answering any user queries, the entire document corpus is encoded with Sentence-BERT (SBERT) [44], TF-IDF, and BM25 features. A user enters a query and it is encoded with a similar combination of featurizers. This query encoding is used to index the featurized documents and thus return the most semantically similar documents to the query. Having retrieved these documents, the next task is ranking for presentation to the user. First, the retrieved documents and query are passed as input to a multi-hop question answering model and an abstractive summarization system. The output from these models are weighted with the original scoring from the retrieval step, and the top scoring documents are presented to answer the query.
  CO-Search is a combination of many cutting-edge NLP models. Their pre-training task for the SBERT encoder is very interesting. The authors train SBERT to take a paragraph from a research paper and classify whether it cites another paper, given only the title of the other paper. SBERT is a siamese architecture which takes one sequence as input at a time. SBERT uses the cosine similarity loss between the output representation of each separately encoded sequence to compare the paragraph and the title. The representation learned from this pre-training task is then used to encode the documents and queries, as previously describe. We will unpack the question answering and abstractive summarization systems later in the survey.

**Fig. 5:**
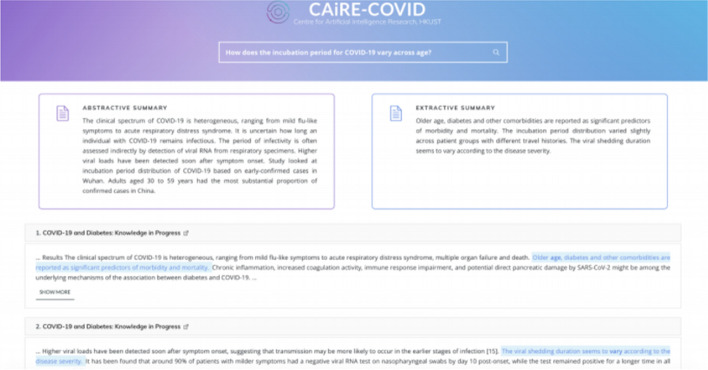
User Interface for the CAiRE-COVID Literature Mining system consisting of Extractive Summary, Abstractive Summary, and most relevant documents (Image taken from Su et al. [39])

The preceding list are examples of Information Retrieval (IR) systems. As mentioned previously, IR describes the task of finding relevant documents from a large set of candidates given a query. These systems typically deploy multi-stage processing to break up the computational complexity of the problem. The first stage is the retrieval stage, where a set of documents much smaller than the total set is returned that best match the query. This first stage of retrieval has only recently integrated the use of neural representations. As previously listed, many systems combine TF-IDF or BM25 sparse vectors with dense representations derived from SBERT. The relationship between these representations is well stated in Karpukhin et al. [33], “the dense, latent semantic encoding of contexts and questions is complementary to the sparse vector representation by design”. This describes how some queries benefit massively from keyword features, whereas others need the contextual information captured in SBERT-style representations.

Deep Neural Networks can be viewed as compression machines for the sake of semantic nearest neighbor retrieval. They take high-dimensional inputs such as the matrix embedding of a paragraph and gradually compress it into a much lower dimensional vector. BERT takes in a sequence of 512 tokens and embeds it into a matrix of size (512 × 768), 768 being the hidden dimension used in the BERT-base release. BERT then processes this matrix through 12 transformer blocks, eventually connecting it to fully-connected layers that end up with an output vector corresponding to each token index. In the BERT architecture, the final vector is indexed at the beginning, [CLS] token. This final vector is the representation of the original sequence of 512 tokens. The SBERT architecture, used in the listed systems, has a more explicit aggregation for the final output vector, rather than indexing the [CLS] token.

In the first stage of Neural Retrieval, we would like to use these vector representations to find the most similar documents to our query. BERT is very successful at pair-wise regression tasks. This is where two sequences are passed in as input, separated by a [SEP] token. The cross-sequence attention in BERT classifies the relationship of the sequences. This is the setup for tasks like Semantic Text Similarity (STS), Natural Language Inference (NLI), and Quora Question Pairs (QQP). However, this setup is costly for information retrieval, passing in each query and document to get a similarity classification would require quadratic comparisons.

Sentence-BERT (SBERT) begins the transition to Transformer-based neural retrieval. SBERT uses a siamese architecture that avoids the pairwise bottleneck of BERT. Siamese architectures describe passing two inputs separately through a neural network and comparing the output representations. Each SBERT “tower” takes a single sequence as input and is trained with a cosine similarity loss. SBERT is then used to encode the documents from a database. Nearest neighbor GPU index optimizations [50] are extremely fast at finding the most similar representations to a query embedding. This is a significant improvement because the semantics contained in these representations are much better than TF-IDF or BM-25 features. The second stage is the refining or re-ranking of initially matched documents. The second stage faces a much smaller set of total documents than initial retrieval and the bottleneck of pairwise models is negligible.

When asking a question about COVID-19, we might not want to be redirected to a list of articles to answer our question. We desire intelligent systems that can directly answer our question. This is the challenge of Question Answering. Tang et al. [51] describe the challenge of constructing datasets for COVID-19 QA in a similar format as the SQuAD dataset. Five annotators working on constructing this dataset for 23 hours resulted in 124 question-article pairs. This includes deconstruction of topics such as “decontamination based on physical science” into multiple questions such as “UVGI intensity used for inactivating COVID-19” and “Purity of ethanol to inactive COVID-19”. The authors demonstrate the zero-shot capabilities of pre-trained language models on these questions. This is an encouraging direction as Shick and Shutze [52, 53] have recently shown how to perform few-shot learning with smaller language models.

#### Knowledge Graph Construction

**Fig. 6:**
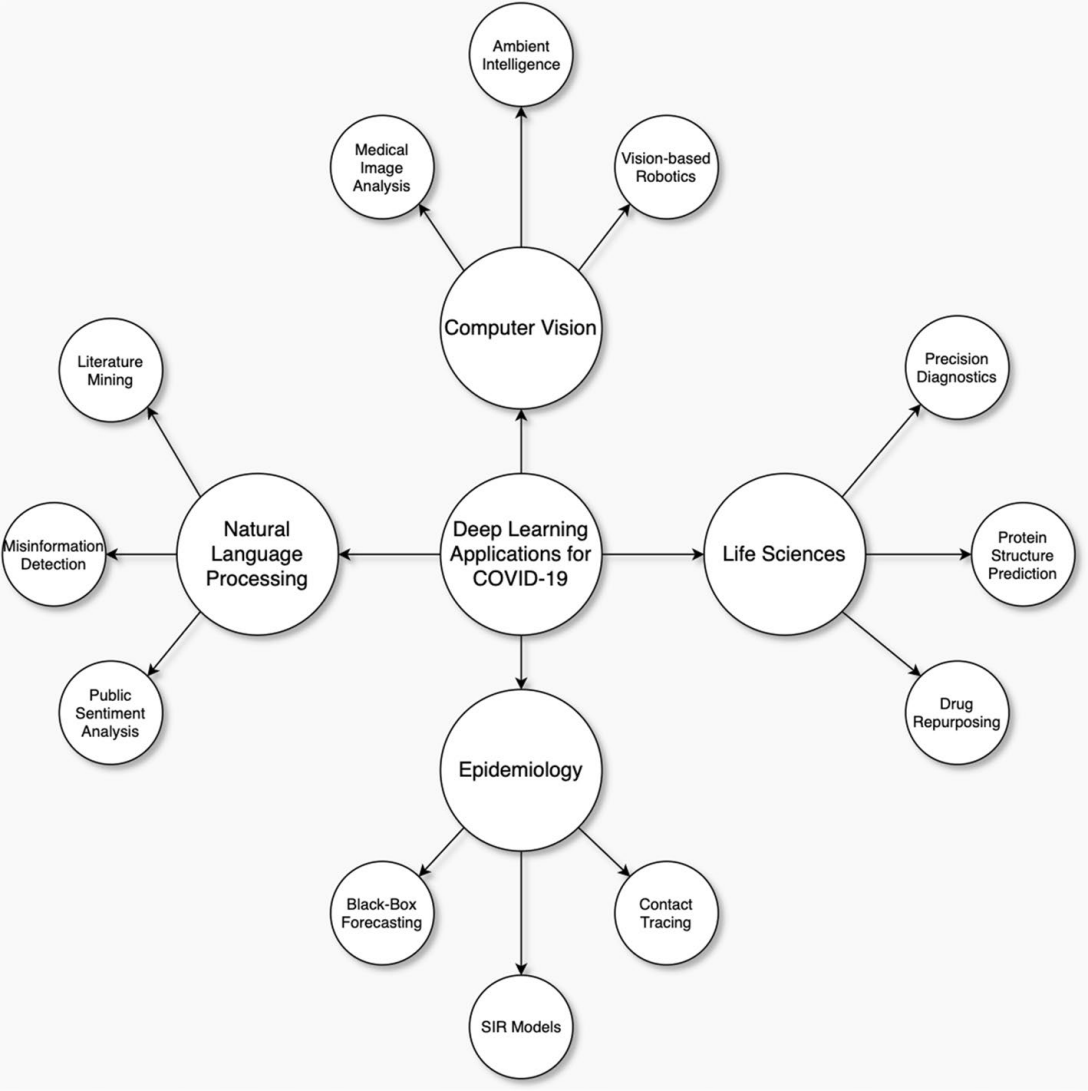
A Knowledge Graph organization of our survey on Deep Learning to fight COVID-19. Here every relation is “A contains B”

One of the best mechanisms of organizing information is the use of Knowledge Graphs. Figure 6 is an example of a Knowledge Graph of our surveyed Deep Learning applications for COVID-19. Each relation in this example is A “contains” B. This is an illustrative example of organizing information topologically. It is often easier to understand how complex systems work or ideas connect when explicitly linked and visualized this way. We want to construct these kinds of graphs with all of the biomedical literature relevant to COVID-19. This raises questions about the construction and usability of knowledge graphs. Can we automatically construct these graphs from research papers and articles? At what scale is this organization too overwhelming to look through?

In application to COVID-19, we would like to construct Biomedical Knowledge Graphs. These graphs capture relations between entities such as proteins and drugs and how they are related such as “chemical A inhibits the binding of protein B”. Richardson et al. [8] describe how they use the BenevolentAI knowledge graph to discover baricitinib as potential treatment for COVID-19. In this section, we focus on how NLP is used to construct these graphs. Under our “Life Sciences” section, we will discuss the potential use of graph neural networks to mine information from the resulting graph-structured data.

**Fig. 7:**
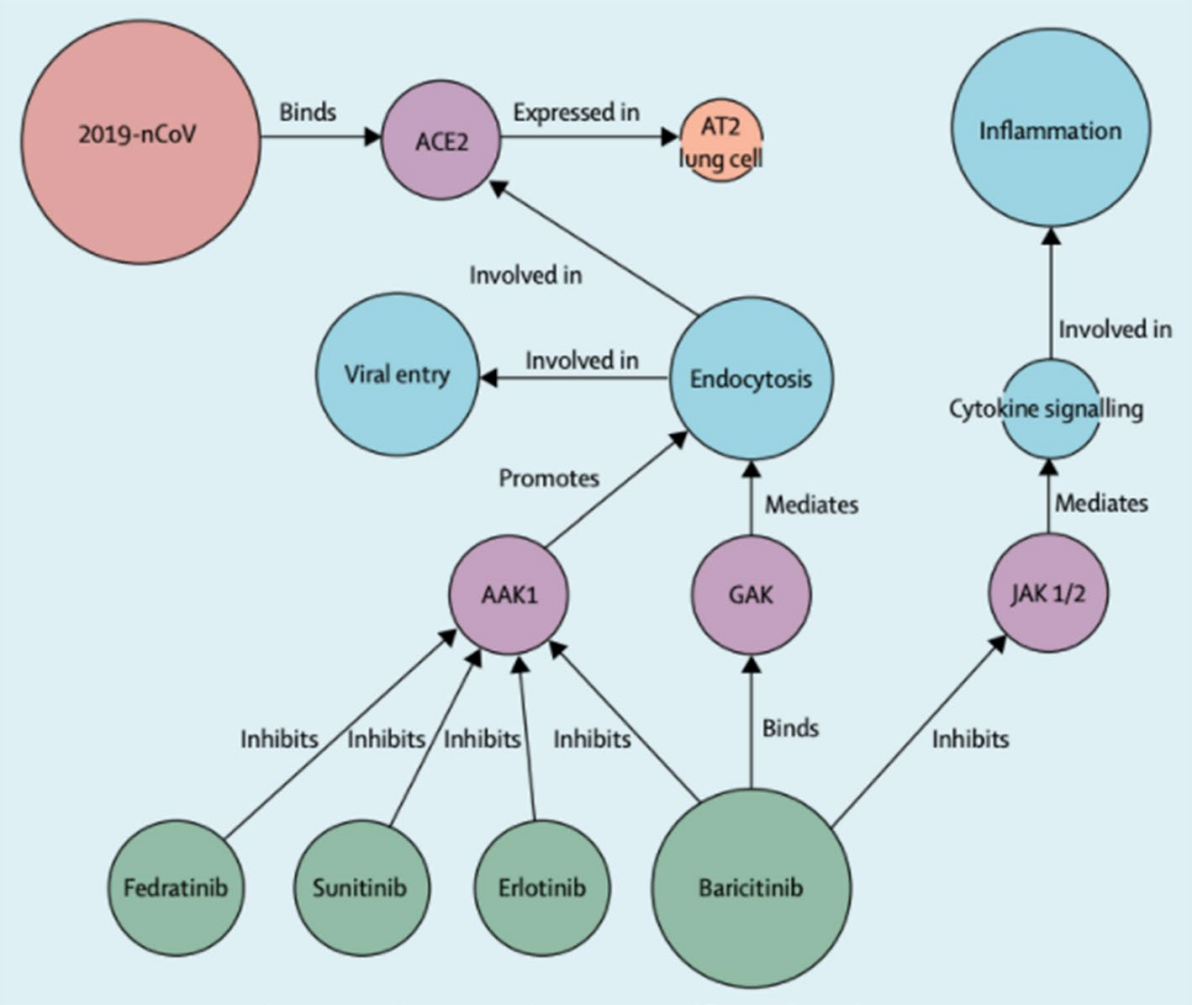
BenevolentAI Knowledge Graph used to suggest baricitinib as a treatment for COVID-19 (Image taken from Richardson et al. [8])

Figure 7 shows examples of different nodes and links. We see the 2019-nCoV node (continually referred to as COVID-19 in our survey), the ACE2 membrane protein node, and the Endocytosis cellular process node, to name a few. The links describe how these different nodes are related such as 2019-nCoV “Binds” ACE2, ACE2 “Expressed in” AT2 lung cell. Richardson et al. [8] describe how this structure allows them to query the graph and form hypotheses about approved drugs. The authors originally have the hypothesis that the AAK1 protein is one of the most known regulators of endocytosis and that disruption of this might stop the virus from entering the cells. The knowledge graph returns 378 AAK1 inhibitors, 47 having been approved for medical use and 6 that have inhibited AAK1 with high affinity. However, the knowledge graph shows that many of these compounds have serious side-effects. baricitinib, one of the 6 AAK1 inhibitors, also binds another kinase that regulates endocytosis. The authors reason that this can reduce both viral entry and inflammation in patients. This is further described in Stebbing et al. [54].

We note that this kind of traversal of the Knowledge Graph requires significant prior knowledge on the part of the human-in-the-loop. This motivates our emphasis on Human-AI interaction in “Limitations of Deep Learning”. Without human accessible interfaces, the current generation of Artificial Intelligence systems are useless. We will describe how Deep Learning, rather than expert querying, can be used to mine these graphs in our section on “Life Sciences”. Within the scope of NLP, we describe how these graphs are constructed from large datasets. This is referred to as Automated Knowledge Base Construction.

A Knowledge Graph is composed of a set of nodes and edges. We can set this up as a classification task where Deep Learning models are tasked to classify nodes in a body of text. This task is known as Named Entity Recognition (NER). In addition to identifying nodes, we want to classify their relation. As a supervised learning task, these labels include the set of edges we want our Knowledge Graph to contain, such as “inhibits” or “binds to”. Deep Learning serves classify nodes and edges according to a human-designed set, not to define new nodes and relations. Describing their system called PaperRobot, built prior to the pandemic outbreak, Wang et al. describe that “creating new nodes usually means discovering new entities (e.g. new proteins) through a series of laboratory experiments, which is probably too difficult for PaperRobot” [55].

The labeled nodes are linked together with an entity ontology defined by biological experts. Defined by Guarino et al., “computational ontologies are a means to formally model the structure of a system, i.e., the relevant entities and relations that emerge from its observation, and which are useful to our purposes” [56]. Wang et al. [57] link the MeSH IDs entities together based on the Comparative Toxicogenomics Database (CTD) ontology. From Davis et al. [58], “CTD is curated by professional biocurators who leverage controlled vocabularies, ontologies, and structured notation to code a triad of core interactions describing chemical-gene, chemical-disease and gene-disease relationships”.

**Fig. 8:**
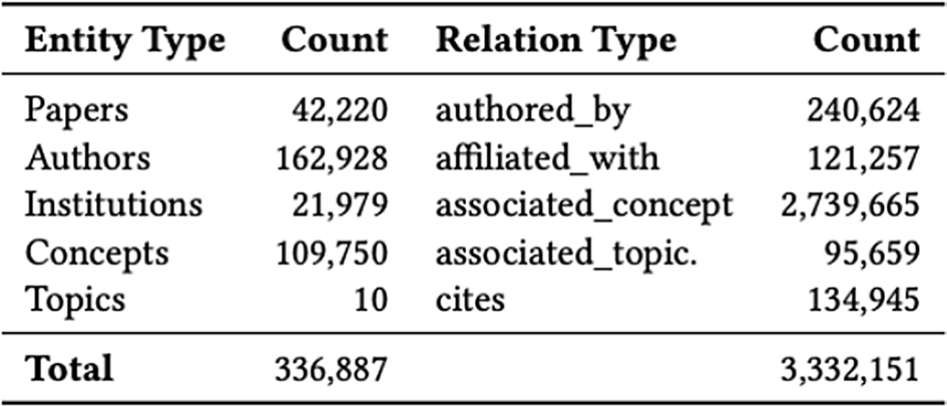
Meta-data on the count of Entities in CKG and Relation information (Image taken from Wise et al. [60])

Wise et al. [59] construct a similar graph containing 336,887 entities and 3,332,151 relations. The set of nodes and edges are shown in Fig. 8. The authors use a combination of graph and semantic embeddings to answer questions with the top-k most similar articles, similar to the Information Retrieval systems previously described. Hosted on http://www.CORD19.aws, their system has seen over 15 million queries across more than 70 countries.

Zeng et al. [61] describe the construction of a more ambitious Knowledge Graph from a large scientific corpus of 24 million PubMed publications. Their graph contains 15 million edges across 39 types of relationships connecting drugs, diseases, proteins, genes, pathways, and expression. From their graph they propose 41 repurposable drugs for COVID-19. Chen et al. [62] present a more skeptical view of NER for automated COVID-19 knowledge graph construction. They highlight that even the more in-domain BioBERT model has not been trained on enough data to recognize entities such as “Thrombocytopenia”, or even “SARSCOV-2”. They instead use a co-occurrence frequency to extract nodes and use word2vec [63] similarity to filter edges.

In SciSight [64], the authors design a knowledge graph that is integrated with the social dynamics of scientific research. They motivate their approach highlighting that most of these Literature Mining systems are designed for targeted search. Targeted search is defined as search where the researchers know what they are looking for. SciSight is designed for exploration and discovery. This is done by the construction of knowledge graph of topics as well as the social graphs of the researchers themselves.

#### Misinformation Detection

The spread of information related to SARS-CoV-2 and COVID-19 has been chaotic, denoted as an infodemic [65]. From conspiracy theories ranging from causal attribution of 5G networks to false treatments and reporting of scientific information, how can Deep Learning be used to fight the infodemic? In our survey we will look at this under the lens of the spread of misinformation and the detection of it. The detection of misinformation has been formulated as a text classification or semantic similarity problem. Our original description of the GLUE benchmark should help readers understand the core Deep Learning problem in the following surveyed experiments.

**Fig. 9:**
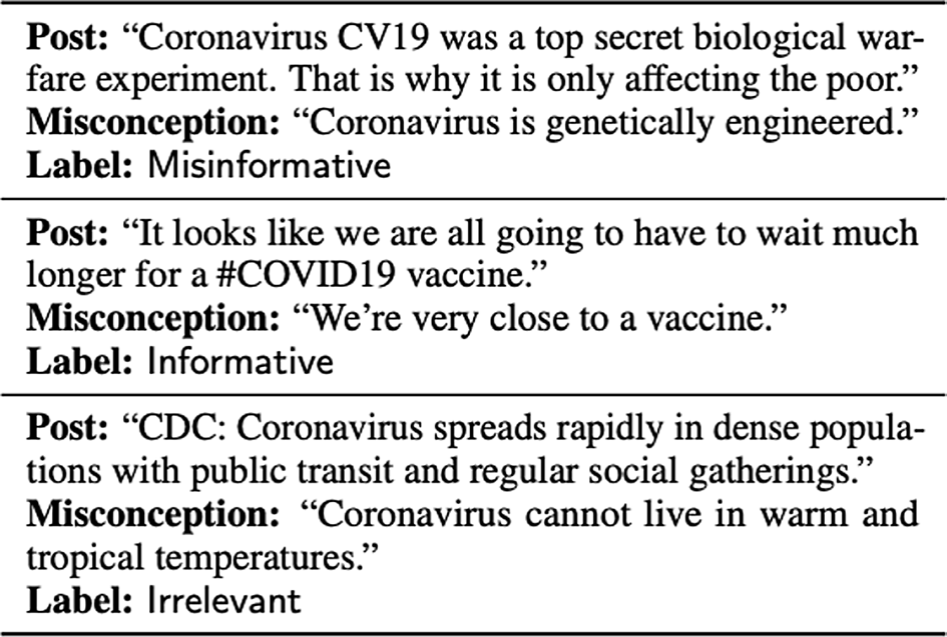
Examples of Misinformation Labels (Image taken from Hossain et al. [69])

Many studies have built classification models to flag tweets potentially containing misinformation. These papers mostly differ in how they label these tweets. Alam et al. [66] label tweets according to 7 question labels; contains a verifiable factual claim, is likely to contain false information, is of interest to the general public, is potentially harmful to a person, a company, a product, or society, requires verification by a fact-checker, poses a specific kind of harm to society, and requires the attention of a government entity. Dharawat et al. [67] look at the seriousness of misinformation, reasoning that “urging users to eat garlic is less severe than urging users to drink bleach”. Their Covid-HeRA dataset contains 61,286 tweets labeled as not severe, possibly severe, highly severe, refutes/rebuts, and real news/claims. Hossain et al. [68] collaborate with researchers from the UCI school of medicine to establish a set of common Misconceptions. These misconceptions are used to label Tweets. Examples of this are shown in Fig. 9.

The detection of misinformation and fact verification has been studied before the COVID-19 infodemic. The most notable dataset of this is the FEVER, Fact Extraction and Verification, dataset [70]. This dataset contains 185,445 claims generated by human annotators. The annotators of the dataset were presented an introduction section of a Wikipedia article and asked to generate a factual claim and then perturb that claim such that it is no longer factually verified. We refer readers to their paper to learn about additional challenges of constructing this kind of dataset [70].

**Fig. 10:**
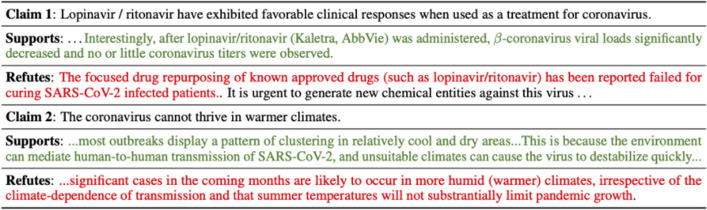
COVID-19 claim examples about COVID-19 and corresponding evidence retrieved (Image taken from Wadden et al. [71])

Constructing a new dataset that properly frames a new task is a common theme in Deep Learning. Wadden et al. [71] construct the SciFact dataset to extend the ideas of FEVER to COVID-19 applications. This is different from classification task formulations previously mentioned in that they are more creative in task design. SciFact and FEVER introduce new datasets that show how supervised learning can tackle Misinformation Detection. Wadden et al. [71] design the SciFact dataset to not only classify a claim as true or false, but to provide supporting and refuting evidence as well. Wadden et al. [71] deploy a clever annotation scheme of using “citances”, sentences in scientific papers that cite another paper, as examples of supporting or refuting evidence for a claim. Examples of this are shown in Fig. 10. The baseline system they deploy to test the dataset is an information retrieval model. We refer readers to our previous section on Literature Mining to learn more about these models.

The authors of SciFACT and FEVER baseline their datasets with neural information retrieval systems. Their systems rely on sparse feature vectors such as TF-IDF and distributed representations of knowledge implicitly stored in neural networks weights. In the previous section we described Knowledge Graphs. Knowledge Graphs for fact verification may be more reliable than neural network systems. We could additionally keep an index of sentences and passages that resulted in relations added to the knowledge graph. This could facilitate evidence explanations. The primary difference with this approach is the extent of automation. Querying Knowledge Base and sifting through relational evidence requires much more human-in-the-loop interaction than neural systems. This approach is also bottlenecked by the problem of scale with evidence. We will continue to compare the prospects of Knowledge Graphs and Neural Systems in our “Discussion” section.

#### Public Sentiment Analysis

The uncertainty of COVID-19 and the challenge of quarantine ignited mental health issues for many people. NLP can help us gauge how the public is faring from multiple angles such as economic, psychological, and sociological analysis. Are individuals endorsing or rejecting health behaviors which help reduce the spread of the virus? Previous studies have looked at the use of Twitter data for election sentiment [72, 73]. This section covers extensions of this work looking into aspects of COVID-19.

Twitter is one of the primary sources of data for Public Sentiment Analysis. Other public data sources include news articles or Reddit. Tweet classification is very similar to the Single-Sentence GLUE benchmark [29] tasks previously described. Each Tweet has a maximum of 280 characters, which will easily fit into Transformer Neural Networks. Compared with Literature Mining, there is no need to carefully construct atomic units for Tweets.

The core question with Twitter data analysis is filtering of the extracted Tweets into categories. This is usually done by keyword matching. Muller et al. [28] use the keywords “wuhan”, “ncov”, “coronavirus”’, “covid”, “sars-cov-2”. Filtering by these keywords over a span from January 12th to April 16th, 2020 resulted in 22.5 M collected tweets. The authors use this dataset to train COVID-Twitter-BERT with self-supervised language modeling. Previously, we discussed the benefit of in-domain data for self-supervised pre-training detailed in [23]. COVID-Twitter-BERT is then fine-tuned for five different Sentiment classification datasets. Two of these datasets target Vaccine Sentiment and Maternal Vaccine Stance. Compared to fine-tuning the BERT [24] model pre-trained on out-of-domain data sources such as Wikipedia and books, fine-tuned COVID-Twitter-BERT models achieve an average 3% improvement across 5 classification tasks. The average improvement was brought down by a very small improvement on the Stanford Sentiment Treebank 2 (SST-2) dataset, which does not consist of Tweets. This further highlights the benefits of in-domain self-supervised pre-training for Natural Language Processing, and more broadly, Deep Learning applications.

Nguyen et al. [74] construct a dataset of 10K COVID tweets. These tweets are labeled as to whether they provide information about recovered, suspected, confirmed, and death cases, as well as location or travel history of the cases, or if they are uninformative altogether. This dataset was used in the competition WNUT-2020 Task 2: Identification of Informative COVID-19 English Tweets. Chauhan [75] describes the efficacy of data augmentation to prevent over-reliance on easy clues such as “deaths” and “died” to identify informative tweets. Sancheti et al. [76] describe the use of semi-supervised learning for this task, finding benefits from leveraging unlabeled tweets.

Loon et al. [77] explore the notion that SARS-CoV-2 “has taken on heterogeneous socially constructed meanings, which vary over the population and shape communities’ response to the pandemic.” They tie Twitter data with Google COVID-19 Community Mobile Reports [78] and find that political sentiment can predict how much people are social distancing. They find that residents social distanced less in areas where the COVID sentiment endorsed concepts of fraud, the political left, and more benign illnesses.

Further exploration into Deep Learning components such as architecture design, loss functions, or activations [79] will not be as important as dataset curation. With a strong dataset, text classification is an easy task for cutting-edge Transformer models. Later on, we will look at the Limitations of Deep Learning that highlight what makes this task challenging from a perfect performance perspective. Another topic in our coverage of limitations is the importance of Human-AI interaction. This is relevant for all applications discussed, but especially for public sentiment with respect to user interfaces. Public sentiment is typically interpreted by economists, psychologists, and sociologists who may not be comfortable adding desired functionality to a PyTorch [80] or Tensorflow [81] codebase. We emphasize the importance of user interfaces that allow users to integrate Public Sentiment Analysis into free-text answers in surveys.

### Computer Vision

Computer Vision, powered by Deep Learning, has a very impressive resume. In 2012, AlexNet implemented a Convolutional Neural Network with GPU optimizations. AlexNet set its sights on the ImageNet dataset. The result significantly past the competition with a 63.3% top-1 accuracy. This marked a large improvement from 50.9% with manually engineered image features. AlexNet inspired further interest in Deep Neural Networks for Computer Vision. Researchers designed new architectures, new ways of representation learning from unlabeled data, and new infrastructure for training larger models. In 2020, eight years after AlexNet, the Noisy Student EfficientNet-L2 model reached 88.4% top-1 accuracy, an absolute improvement of 25.1%. The Deep Computer Vision resume continues with generation of photorealistic facial images, transferring artistic style from one image to another, and enabling robotic control solely from visual input.

The success of Deep Computer Vision is largely attributed to the ImageNet dataset [82]. The ImageNet competition is a dataset that contains 1.2 million images labeled in 1,000 categories. Images are inputted to Deep Neural Networks as tensors of the dimension height × width × channels. For example, most ImageNet images have the dimension 128 × 128 × 3 pixels. The resolution of image inputs to Deep Learning is an important consideration for the sake of computational and storage cost.

Computer Vision stands to transform Healthcare in many ways. The most salient and frequently discussed application to COVID-19 is medical image diagnosis. Deep Learning has performed extremely well at medical image diagnosis and has underwent clinical trials across many diseases [83]. Medical image tasks mostly consider classification and segmentation, as well as reconstruction from 2-D slices in a CT-scan to make up the final 3-D view.

Computer Vision also stands to aid in subtle hospital operations. Haque et al. [10] describe “Ambient Intelligence” where Computer Vision aids in physical therapy to combat ICU-acquired weakness, ensures workers and patients wash their hands properly, and improves surgical training, to name a few. Computer Vision equips cameras to “understand” what they are recording. They can identify people. They can label every pixel of a road for the sake of self-driving cars. They can map satellite images into road maps. They can imagine what a sketch drawing would look like if it was a photographed object. Here we use “understand” for the sake of hyperbole, really meaning that it can answer semantic questions about image or video data. These applications describe the potential of Computer Vision to enable a large set of subtle applications in pandemic response, such as face mask detection and monitoring social distancing or hospital equipment inventory. We hope to excite readers about the implications and gravity of this technology; however, in “Limitations of Deep Learning” we highlight “Data Privacy” issues with this kind of surveillance.

Economic damage is one of the greatest casualties of COVID-19. How can we engineer contact-free manufacturing and supply chain processes that don’t endanger workers? Vision-based robotics is a promising direction for this. Current industrial robotics rely on solving differential equations to explicitly control desired movement. This works great when the input can be extremely controlled, but breaks down when the robot must generalize to new inputs. Vision-based robotics offers a more general control solution that can adapt to novel orientations of objects or weight distributions of boxes.

#### Medical Image Analysis

Assisting and automating Medical Image Analysis is one of the most commonly discussed applications of Deep Learning. These systems are improving rapidly and have moved into clinical trials. We refer readers to Topol’s guidelines for AI clinical research, exploring lessons from many clinical fields [84]. Our coverage of Medical Image Diagnosis for COVID-19 is mostly focused on classification of COVID-19 pneumonia, viral pneumonia, or healthy chest radiographs. There are many studies that focus on the Semantic Segmentation task, where every pixel in an image is classified. We discuss the Semantic Segmentation application in our section on “Interpretability” with respect to improving Human-AI Interaction. In our analysis, chest radiographs are sourced from either X-ray imaging or higher-resolution CT scans. Motivating the use of radiograph diagnosis, Fang et al. [1] find a 98% sensitivity with CT detection compared to 71% with RT-PCR. Ai et al. [85] look at the correlation between Chest CT and RT-PCR testing for 1014 patients concluding that “chest CT may be considered as a primary tool for the current COVID-19 detection in epidemic areas” [85]. Das et al. [86] highlight some reasons to prefer chest X-rays over CT-scans, namely that they are cheaper and more available, and they have lower ionizing radiation than CT scans do.

In our section on “Learning from Limited Labeled Datasets”, we will further discuss the challenge of fitting Deep Learning models with relatively small datasets. Medical image analysis may be the best example of this. Compared to the 1.4 million images in ImageNet, we rarely have more than 1000 COVID-19 positive chest radiographs. This is especially important in a pandemic outbreak situation, where we need to gather diagnostic information as fast as possible. Researchers have turned to variants of supervised learning as the solution to this problem. This includes transfer, self-supervised, weakly supervised, or multi-task learning.

Fortunately for this application, there are plenty of existing datasets that seek to classify pneumonia from Chest radiographs and can be used to bootstrap representations learned for COVID-19 detection. Irvin et al. [7] constructed the Chexpert dataset of 224,316 chest radiographs of 65,240 patients prior to the COVID-19 outbreak. It is important to differentiate between COVID-19 and viral pneumonia, rather than thinking about this problem through the lens of COVID-19 vs. healthy. For an example of the dataset sizes available, Wenhui et al. [87] train their model on a dataset of 1341 normal, 1345 viral pneumonia, and 864 COVID-19 images. Wang et al. [42] published the COVIDx dataset with 13,975 CXR images across 13,870 unique patients. However, this dataset only contains 358 CXR images from 266 COVID-19 patient cases. We refer readers to [88–90] to review common approaches to Deep Learning with class-imbalanced datasets.

Our literature review reveals that many of these studies pre-train models on the ImageNet dataset and then fine-tune them on the COVIDx dataset This representation learning strategy is known as transfer learning. Farooq and Hafeez [91] transfer weights from a pre-trained ResNet50, Wang et al. [92] explore the Inception model, and Afshar et al. [93] deploy a Capsule Network. We do not report performance metrics such as accuracy, precision, or recall reported in these papers due to experimentation with extremely small datasets. All of these papers report gains with transfer learning compared to random weight initializations.

Raghu et al. [83] report a more sobering view of Transfer Learning for Medical Image Analysis. Most notably, they show that as medical imaging tasks collect more data, there is little to no benefit in ImageNet pre-training. On larger datasets such as Retina and ChexPert, there is no significant difference in performance when using Transfer Learning. However, Transfer Learning does improve performance in the small data regime. Small data is defined in this study as 5000 images. In the interest of COVID-19 applications, we note that this small data benefit is extremely important. However, If COVID-19 continues to spread and more positive cases are collected, we do not expect transfer learning from ImageNet to continue to be useful. We note this is heavily related to the domain mismatch between ImageNet and chest radiographs. We do expect transfer learning to continue to be effective from datasets such as CheXpert [7].

Transfer learning usually describes supervised learning on one dataset, and then using these weights to initialize supervised learning on another dataset. Transfer learning can also refer to using self-supervised learning on one dataset, or other learning variants we mentioned in our Introduction. There have been many promising advancements in self-supervised representation learning. For our COVID-19 applications, we will consider the use of contrastive self-supervised learning. This learning algorithm pushes representations of positive pairs to be close together and negative pairs far apart. The emerging practice in contrastive self-supervised learning [94, 95] is to form positive pairs from views of an image, where a view is a data augmentation transformation of the image [96]. MoCo [94] and SimCLR [95] are two of the most promising models for this. MoCo is generally preferred due to memory efficiency.

Zhang et al. [97] take a unique multi-modal approach to construct this learning task. They look to the short text descriptions that are paired with medical images. These annotations are much less detailed than the high-quality annotations that bottleneck the data collection process. Researchers have looked at how these text descriptions can facilitate visual representation learning, but rule-based label extraction is often inaccurate and limited to a few categories, and more generally these rules are domain-specific and sensitive to the style of text. Zhang et al. leverage this text to form image-text pairs with the medical images, naming their algorithm Contrastive Visual Representation Learning from Text (ConVIRT).

**Fig. 11:**
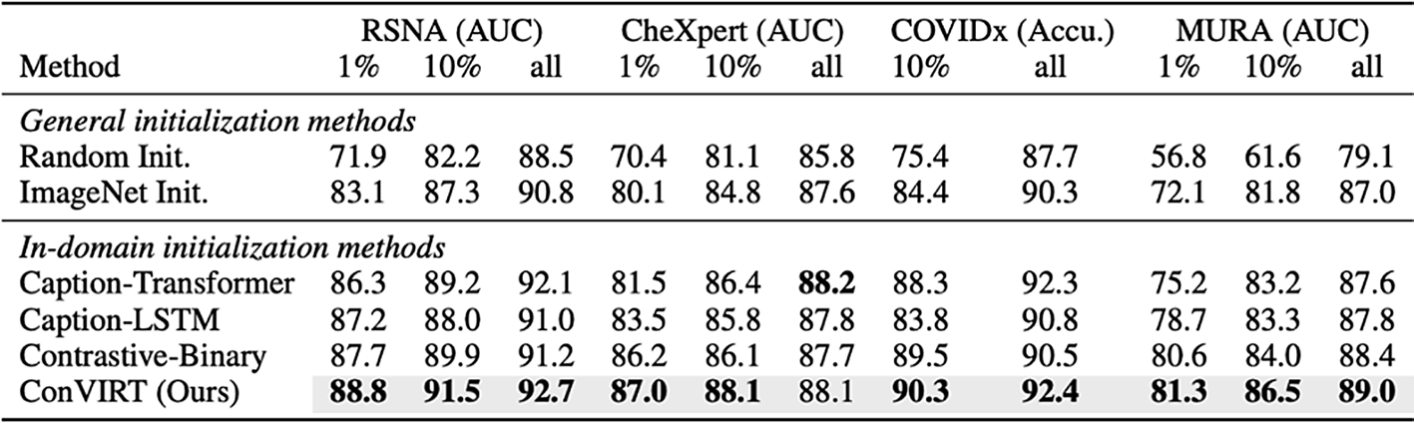
AUC and Accuracy performance gains from ConVIRT (Image taken from Zhang et al. [97])

The improvements of fine-tuning ConVIRT on different medical image datasets, such as COVIDx, compared to ImageNet initialization and other techniques is shown in Fig. 11. In the setting with 1% of the CheXpert labels, transfer learning from ConVIRT achieves an AUC of 87.0 compared to 80.1 from ImageNet pre-training with supervised learning. With 10% of the COVIDx data, ConVIRT achieves 90.3% accuracy compared to 84.4%. Although more promising than ImageNet transfer learning with supervised learning, with more labeled data, even ConViRT begins to show modest gains to random initialization. Sowirijan et al. [98] also present massive gains of the MoCo representation learning scheme. However, they only report results on the CheXpert dataset.

Even in their takedown of ImageNet-based transfer learning for Medical Image Analysis, Raghu et al. [83] note transfer performance differences by redesigning computational blocks in the ResNet. As mentioned previously, architecture design is one of the most promising trends in Deep Computer Vision research. Subtle architectures changes such as the arrangements of Skip-connections in DenseNet [99] can have a significant performance gain over a more standard ResNet [100] model. Researchers have turned to parameterizing a discrete search space of candidate architectures and searching through it for a suitable architecture. This search is usually done with evolutionary search [101] or reinforcement learning [102]. Wang et al. [42] turn to this research in the development of their COVID-Net for chest X-ray diagnosis. The authors design a macro, high level architecture structure, and a micro set of operations such as 7 × 7 vs. 1 × 1 convolutions, and combine them using a generative synthesis search algorithm. This results in a 3% accuracy improvement over a more standard ResNet-50 architecture.

From our literature review, we believe that representation learning schemes and architecture design are the most salient areas for exploration. We additionally believe that multi-task learning with Semantic Segmentation could improve representation learning. However, multi-task learning can be challenging to implement due to conflicting gradient directions with updates. Collecting labeled data for training Semantic Segmentation models can be extremely tedious as well. Shan et al. [103] present an interesting human-in-the-loop strategy for labeling the pixels in COVID-19 CT scans. We also refer readers to an investigation from Gozes et al. [104] that uses Medical Image Analysis to explore COVID-19 disease progression over time. Finally, we think there may be value in exploring differential diagnosis with the supervised learning label space. This describes using tree structured labels. These labels reward the model if at least recognizes a parent node with the more specific, ground truth diagnosis. This strategy is generally known as Neural Structured Learning [105].

#### Ambient Intelligence

When we think of the intersection of AI and Healthcare or Medicine, we may jump to groundbreaking protein structure prediction or language models that guide research directions. However, not all problems are so grandiose in mission. How many of Healthcare’s problems are subtle day-to-day operations that can be automated? One of the most frequent storylines of the COVID-19 pandemic was fear of an overloaded health system. Here, we look at how Computer Vision enables Ambient Intelligence. Ambient Intelligence is defined by Haque et al. [10] as “physical spaces that are sensitive and responsive to the presence of humans”. This section is a brief summary and reflection of their survey.

**Fig. 12:**
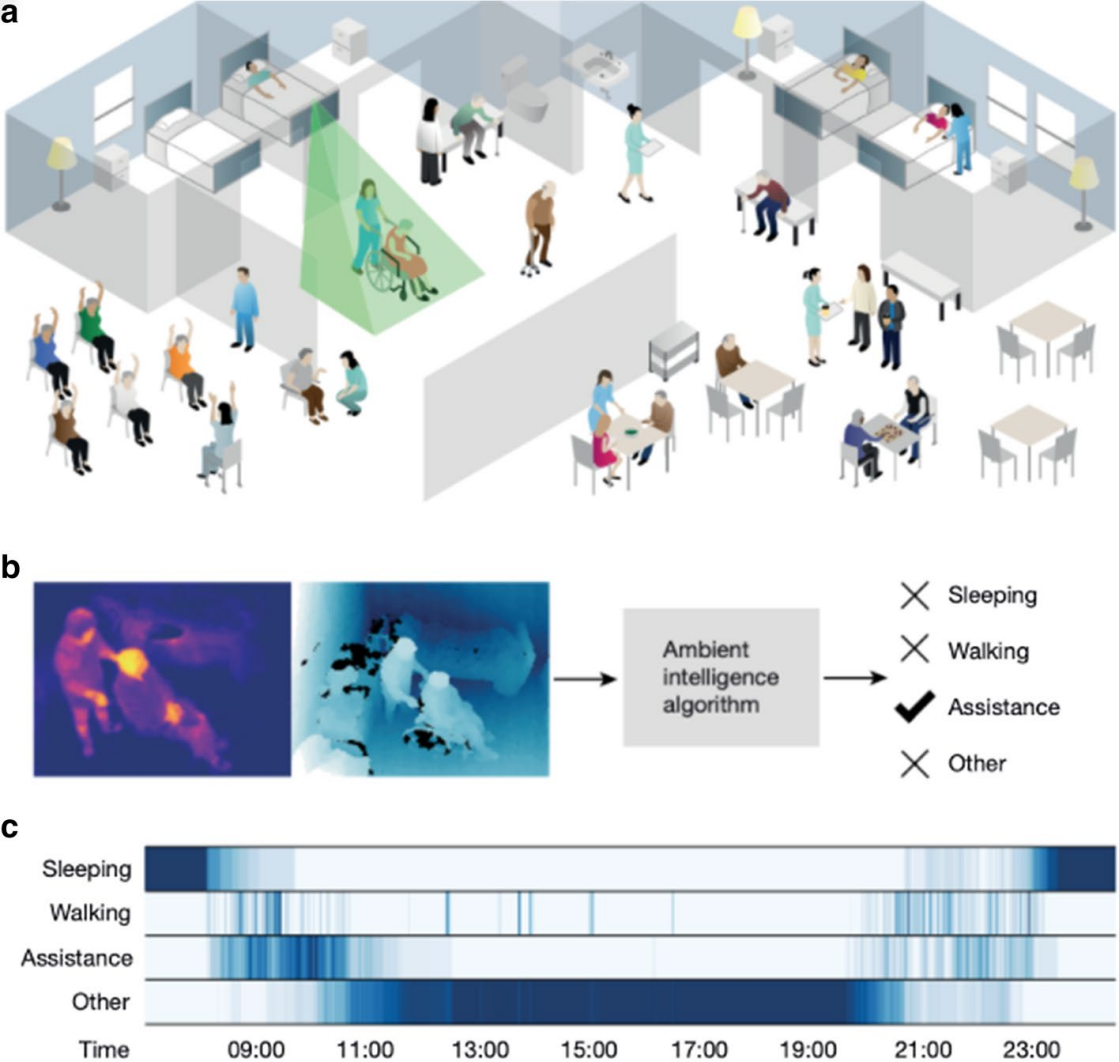
An illustration of Ambient intelligence of daily living spaces in an elderly home equipped with one ambient sensor (Image taken from Haque et al. [10])

The survey begins by describing that decision-support systems have corrected sub-optimal diagnostic and treatment decisions, but automated decisions about physical actions remain esoteric and unexplored. In a report on medical error, Mackary and Daneil found that “as many as 400,000 people die every year in the United States owing to lapses and defects in clinical decision-making and physical actions” [106]. The idea of Ambient Intelligence is to integrate smart sensors that can monitor and collect data about these physical actions. Haque et al. [10] explore physical therapy assistance, hand washing, and surgeon performance evaluation. Figure 12 illustrates the layout of an elderly care unit with caregivers clothed in blue or green. This graphic portrays the scenario where there are more patients than workers, and it may be challenging to record exactly what kind and how much assistance is given. This figure describes a study where a depth and thermal sensor, (shown as a green highlight), are used to observe 1690 activities and 231 instances of caregiver assistance over the span of 1 month. A convolutional neural network trained on this data achieved 86% accuracy at detecting assistance. This automated analysis can record the operations of the elderly care unit without requiring additional overhead on the caregivers.

In application to COVID-19, we are mostly interested in this kind of reporting in Intensive care units (ICUs). Haque et al. [10] describe activity monitoring with Computer Vision to better understand ICU-acquired weaknesses. In a comparison of 224 weak and non-weak patients, Hermans et al. report a lower likelihood of weaning from mechanical ventilation and hospital discharge amongst weak patients, as well as higher in-hospital costs and 1-year mortality. The solution provided by Ambient Intelligence would be to monitor mobility activities to better understand how to combat ICU-acquired weaknesses.

One of the most promising research directions for Deep Learning in Ambient Intelligence will be compression. We need to compress these Deep Neural Networks so that they can be cost-effectively embedded in smart sensors. Some of the most promising approaches to compression include mixed precision, quantization, and pruning. Mixed precision describes using either 2, 4, 8, or 16-bit precision for neural network weights, rather than 32-bits. Quantization is a more effective strategy for reducing this precision. This describes clustering at common values, rather than just truncating to meet precision requirements. Many of the weights in a neural network can be pruned away, or set to zero, and not impact the performance of the model. The lottery ticket hypothesis [107] showed that it is even possible to train pruned networks, rather than waiting until training is completed to prune networks to zero. However, Deep Learning hardware is only recently catching on to accelerate sparse networks, with recent advancements such as Block-Sparse Kernels. Compression is an exciting field of Deep Learning research. Some interesting directions include understanding the success and phenomena of sparse networks, and the opportunity to further explore quantization noise during training [108].

#### Vision-based robotics

Robotic Control is one of the most popular applications and research areas of Deep Learning. As a Deep Learning task, this involves mapping from pixel and sensor states to motor force actions. In this section we are focused on the integration of visual input to the state representation for control. This mapping can be learned through either supervision with demonstrations or reinforcement learning from scratch. An illustrative example of vision-based robotics is the Wozniak coffee cup test [109]. This describes whether a robot would be able to walk into any room and make a cup of coffee. We note that this level of generalization is unnecessary for most applications for COVID-19. We discuss this further in Generalization Metrics.

The range of robotic applications considered varies enormously in the complexity of manipulation required. For example, a robot unleashing a disinfecting spray on a room does not need to have fine-grained manipulation skills. However, a robot assisting a patient with a respiratory does. In a similar way, robots facilitating manufacturing and economic activity vary along this scale as well. Sorting bottles requires more vision than dexterity, whereas assembly requires incredibly dexterous manipulation.

Barfoot et al. [110] outline a plan for the future of Canadian robotics in response to the Pandemic. The authors highlight application potential in disinfection, remote triage, logistics, and delivery. The authors claim that “the pandemic has become an inflection point for accelerating investment in robotics” [110]. Murphy et al. [111] survey 262 reports on ground and aerial robots for the COVID-19 response. They divide robotics applications into categories of Public Safety, Clinical Care, Continuity of Work and Education, Quality of Life, Laboratory and Supply Chain Automation, and Non-Hospital Care.

RoboNet [112] is an open database for visual robotic experience containing 15 million video frames across 7 different robot platforms. Srinivas et al. [113] recently showed a breakthrough performance by introducing a multi-task self-supervised learning system to facilitate visual representation learning. Wu et al. [114] demonstrate how a Soft Actor Critic agent can learn to fold cloth material. We refer interested readers to “The Ingredients of Real-World Robotic Reinforcement Learning” by Zhu et al. [115].

### Life Sciences

This section will address an absolutely massive scope, generally defined here as “Deep Learning for Life Sciences”. Our scope ranges from improving the COVID-19 diagnostic capabilities of blood testing [116] to ground-breaking applications in protein modeling and drug repurposing.

There is no shortage of big data at the intersection of Biology and Deep Learning. Humans contain 45,000 genes and the entire Human Genome contains 3 billion base pairs [117]. There are an estimated 37.2 trillion cells in a human body [118]. These numbers illustrate “Large-Scale Biology” [117]. Despite a solid foundation of information in biology, we do not have an exact model of every physiological pathway in the human body. We cannot exactly understand what will happen with the introduction of a new molecule. However, we can still model proteins such as membrane proteins the virus binds with. These protein models allow us to design potentially inhibiting drugs much better than random chance.

#### Precision Diagnostics

The gold standard test for SARS-CoV-2 has been Reverse Transcriptase-Polymerase Chain Reaction (RT-PCR). RT-PCR is a nucleic acid amplification test that works by iteratively heating up and denaturing the DNA, binding primers, and then attaching enzymes to amplify the total amount of DNA by providing a complement strand. This amplification enables fluorescent probes to highlight the presence of the viral RNA, which would be nearly impossible to do with the pre-amplified sample.

**Fig. 13:**
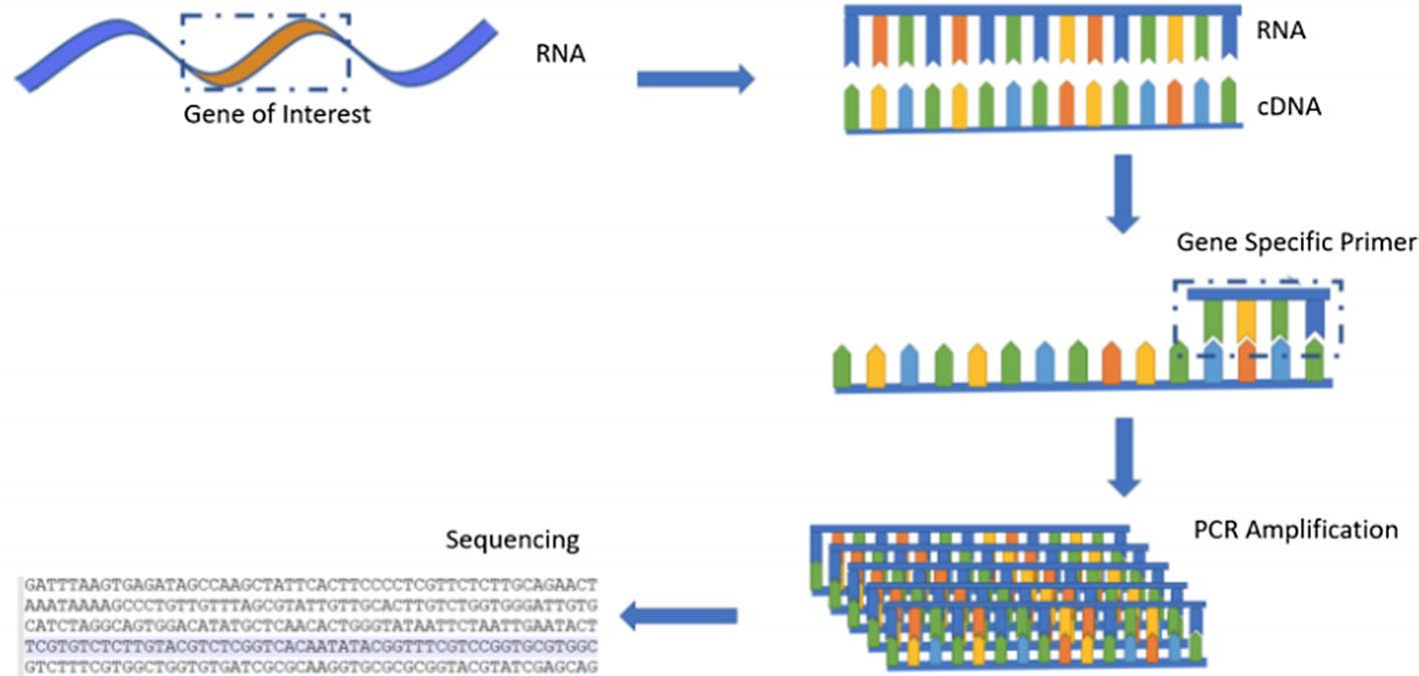
Rough overview of RT-PCR amplifications (Image taken from Lopez-Rincon et al. [119])

Lopez-Rincon et al. [119] highlight the challenge of declaring that the presence of a gene such as ORF1ab evidences SARS-CoV-2 infection. A high-level illustration of RT-PCR testing is shown in Fig. 13, the challenge is correctly classifying the presence of SARS-CoV-2 proteins in the amplified sequence. The authors construct a dataset of viral RNA sequences that are known to be hosted in humans. They encode base pairs ‘A’,’C’,’G’,’T’ into numeric values of 0.25, 0.5, 0.75, and 1. This is opposed to the embedding encodings used for tokens in NLP. Their system achieves 98.75% accuracy in classifying a dataset of 553 coronavirus sequences ranging from 1260 to 31,029 base pairs.

Shiaelis et al. [120] deploy Computer Vision in single-particle fluorescence microscopy imaging to detect SARS-CoV-2. We refer readers to our section on Computer Vision for a description of how Computer Vision problems commonly use Deep Learning. Shiaelis et al. train their model on lab-grown viral images. The authors note that the fluorophore distribution over the surface of different viruses such as size and shape are perfectly fitted for feature extraction with Deep Convolutional Neural Networks. After the domain knowledge used to preprocess, tag, and label these images, they appear surprisingly simple. The authors report different binary classification accuracies between virus strains such as SARS-CoV-2 vs. influenza A strains on lab-grown training sets. Further, the authors have tested their system on about 60,000 clinical samples. This approach claims to take less than five minutes and is a promising step towards rapid mass testing.

**Fig. 14:**
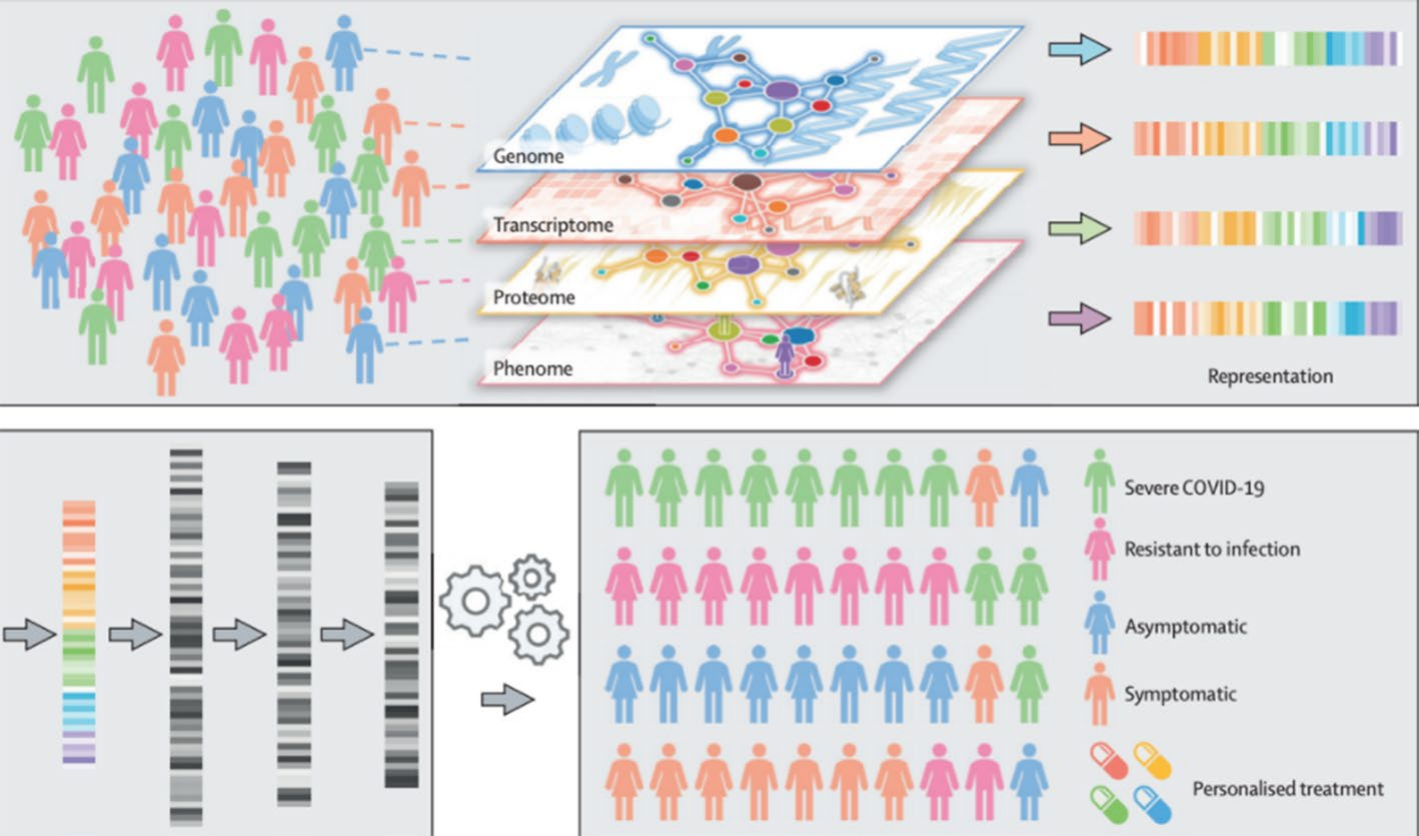
AI for Precision Medicine (Image taken from Zhou et al. [122])

Given some of the general shortcomings of RT-PCR testing such as long turnaround times and availability of tests, researchers have also explored the diagnostic capability of routine blood tests [116]. These blood tests yield data about white blood cell counts, platelets, and plasma levels. Brinati et al. [116] collect this data from 279 patients and achieve a range of 83% to 89% accuracy with a modified random forest classifier. This range is due to different data splits used to evaluate the model. [69] look to combine these features with CT scans and clinical information through a late fusion model. [121] also look at combining a white blood cell test with CT images for diagnosis. Zhou et al. [122] imagine the integration of genomic, transcriptomic, proteomic, and phenomic profiles for a more personalized treatment in drug repurposing. We will describe this application later on the survey. We refer readers to the illustrative diagram from their study [122] describing the integration of different data sources for Precision Diagnostics, shown in Fig. 14. We additionally refer readers to a survey on big data in health informatics from Herland et al. [123] to develop intuition on data available for Precision Diagnostics.

#### Protein structure prediction

**Fig. 15:**
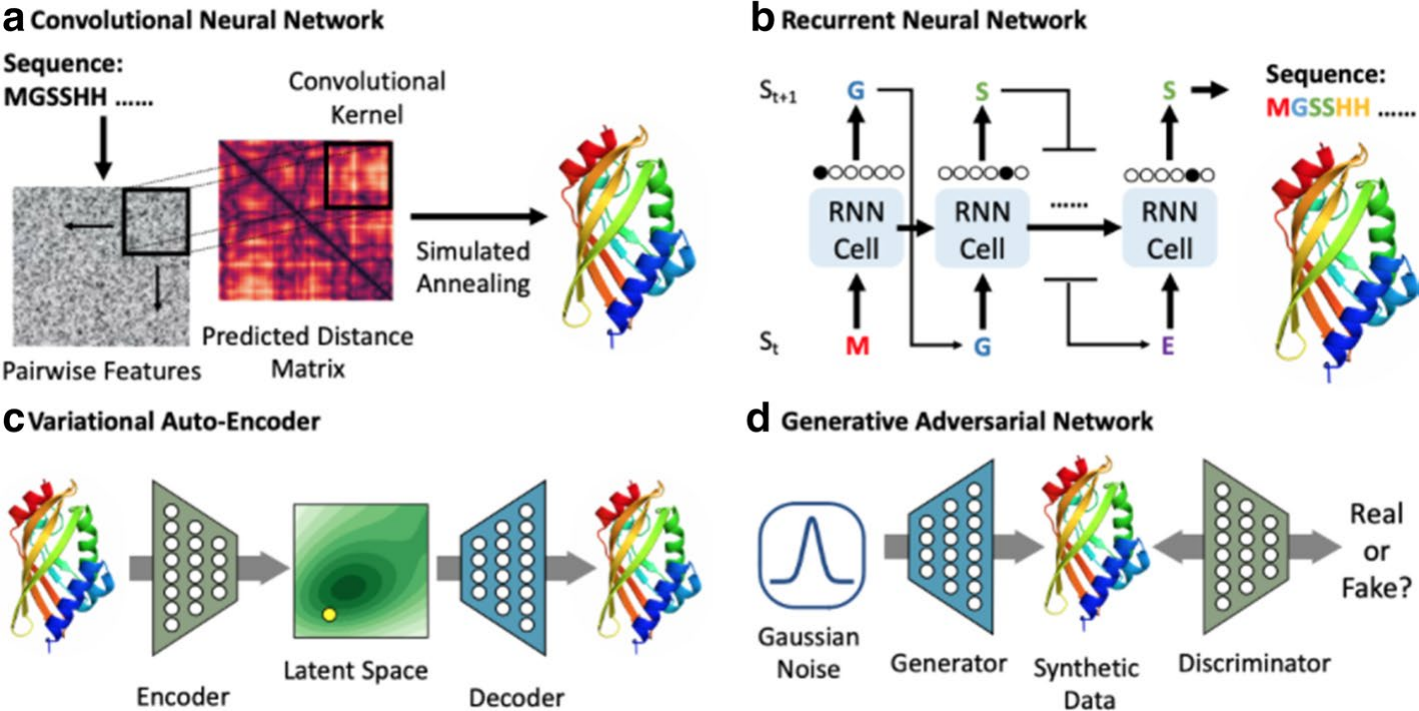
Illustrations of how different Neural Network architectures have been applied to Protein Structural Modeling (Image taken from Gao et al. [127])

One of the keys to understanding the biology of SARS-CoV-2 is the structure of the outer shell proteins. Structure determines the role and function of a protein [124]. Understanding this structure can help with finding potential treatments. “If a group of viruses shares a common protein structure, then therapies for one viral infection can be repurposed for new diseases like COVID-19” [125]. Experimental verification of structure is done with X-ray crystallography, nuclear magnetic resonance imaging, or cryo-electron microscopy. However, this is costly and time consuming. Therefore, researchers are interested in models that can predict this structure. These validated structures have been placed in the Protein Data Bank. This dataset of 1-D amino acid sequences and their resulting, verified 3-D structures is a great starting point for Deep Learning. Figure 15 illustrates some neural network designs for different perspectives on protien structure prediction. From our perspective on understanding Deep Learning problems, the Protein Data Bank is a dataset that can be used with Semi-Supervised Learning. Semi-Supervised Learning describes the learning problem where there is a small, labeled dataset and a larger, unlabeled dataset. There are only 158 K experimentally determined structures available in the Protein Data Bank (PDB). However, there are over 180 M protein sequences recorded in UniProt. These 180 M sequences do not have labeled 3-D structures.

Rato et al. [126] inspire exploring into these un-labeled protein sequences, “billions of years of evolution have sampled the portions of protein sequence space that are relevant to life, so large unlabeled datasets of protein sequences are expected to contain significant biological information” [126]. Rao et al. recognize protein representation learning as a semi-supervised learning problem and introduce the Tasks Assessing Protein Embeddings (TAPE) benchmark. These tasks test the strength of semi-supervised protein representation learning and finds self-supervised pre-training to be effective. We will discuss self-supervised learning further in Learning with Limited Labeled Data.

An important distinction to make in protein structure prediction is between Template and Free Modelling. Template Modeling looks for the most similar sequence that has an experimentally verified 3-D structure, noting that similar amino acid sequences likely have similar 3-D structures as well. From the perspective of Deep Learning, Template Modeling can be thought of as a nearest neighbor problem. We look for the most similar sequence in which we have a verified experimental structure available. Free modeling is the more ambitious task of predicting structure without this kind of reference.

The CASP competition is the premier competition for protein structure prediction [128]. In 2018, AlphaFold [124] placed first in the Free Modeling category with the use of Deep Learning. AlphaFold marked an incremental improvement in the approach to this problem. Traditional models, as well as AlphaFold, use Multiple Sequence Alignment (MSA) to find similar sequences to the sequence to be predicted. These similar sequences are concatenated to the input representation. This input is then mapped to predicting contacts between the carbon atoms of 2 amino acid residues. This contact prediction is traditionally setup as a binary classification problem, do they lie within a given distance? AlphaFold rather predicts this distance directly as a regression problem. This precise modeling required a stronger model, and AlphaFold uses a variant of ResNet [100], developed for Computer Vision, to step up to the challenge.

This has not been game changing in the same sense of AlexNet and ImageNet, but it is a very promising step forward. AlphaFold predicts pairwise distances between amino acids in the 3-D structure, aggregates the resulting potential mean force deterministically, and thus ranks and presents the most likely structure with the lowest energy. Our Discussion section aims to bridge different applications under commonalities of Deep Learning problems. We hypothesize a potential intersection between MSA features and trends in Information Retrieval in our Discussion.

#### Drug repurposing

When discussing Natural Language Processing applications, we looked at how NLP can aid in the construction of Knowledge Graphs (KGs). These KGs can be used to find suitable candidates for drug repurposing. In this section we will explore how we mine this graph-structured biomedical information. Network Medicine [129] is an area of research that looks at the holistic view of interaction networks such as protein-protein or drug-target. This is extremely important because some drugs may look promising on a cellular assay, but show little benefit in real clinical trials. This kind of information can be mined from the biomedical literature and clinical trial reports. A systematic screening of all approved drugs is a promising direction for new treatments. Medicines designed for one disease finding use in another.

Zhou et al. [122] published a comprehensive survey on “Artificial intelligence in COVID-19 drug repurposing”. This survey looks at mining Knowledge Graphs to find FDA-approved drugs that may be suitable for treating COVID-19. The survey also considers combinations of drugs such as baricitinib and remdesivir. The survey primarily reviews research that has used graph representation learning for downstream link prediction. Graph representation learning is a branch of Deep Learning that works with graph-structured data, such as the Knowledge Graphs previously described.

A citation graph is a great way to develop intuition for Deep Learning on graph-structured data. Each paper has an embedding for the text content and it is connected to other papers based on outgoing and incoming citations. Graph inputs to Deep Learning typically consists of two matrices. An adjacency matrix represents connections between the nodes in the graph. The other input matrix is an embedding table with features for each of the nodes. A common layer for processing these features is the Graph Convolutional Layer. This describes learning weights to propagate features along edges in the graph such that message passing only occurs between neighbors. Through several layers, distant neighbors can share information. At the final representation of the network, indexed positions of a node or edge in the feature graph is used for classification. In drug repurposing, this task is commonly link prediction between protein target and drug nodes. We refer interested readers to Hamilton et al. [130] for a more detailed survey on representation learning on graphs.

**Fig. 16:**
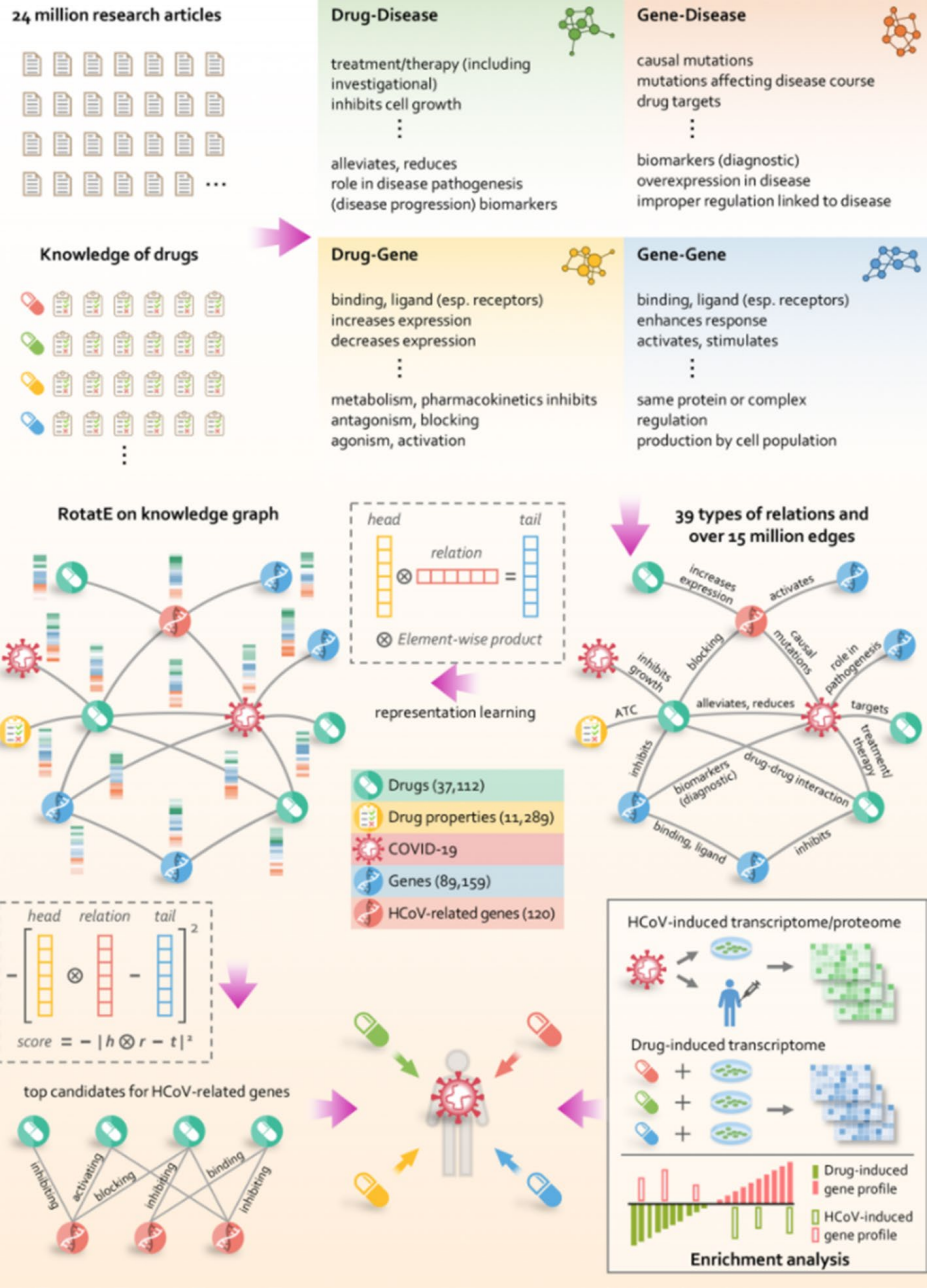
Illustration of the use and construction of CoV-KGE for drug repurposing (Image taken from Zeng et al. [61])

Under the umbrella of Knowledge Graphs, we presented how Zeng et al. [61] constructed a Knowledge Graph with 15 million edges from a corpus of 24 million PubMed publications. From their graph, Zeng et al. [61] use the RotatE graph representation learning algorithm to embed relations into a low-dimensional vector space. They then suggest potential drug candidates by taking the top-k neighbors in the embedding space. The full pipeline is visualized in Fig. 16. We refer readers to [131] for more information on these interaction networks such as Protein-Protein interaction networks.

### Epidemiology

The biggest intervention to the spread of COVID-19 that has been implemented is sheltering-in-place. The solution of limiting contact between people significantly reduces the spread and prevalence of SARS-CoV-2. However, sheltering in-place comes at a massive economic and mental health cost. The field of Epidemiology aims to answer questions about the spread of infectious diseases such as COVID-19. The tools of Epidemiology, such as the SEIR differential equations, help us understand many important questions about the virus. How long will we need to remain quarantined in our homes? How much will quarantining slow down the spread of the virus? What do we know about the Infection rate? Answering these questions is extremely important for allocating scarce resources such as ventilators, personal protective equipment, and ICU beds, as well as for public information. This section will explore how Deep Learning can improve spread forecasting.

#### Black-box spread forecasting

In this section, we will look at different levels of granularity for spread forecasting. This granularity differs with respect to region modeling, such as country- compared to county-wide forecasting, as well as assumptions about the population in the model. We will start with Deep Learning approaches that treat the virus and population as a black-box phenomenon and only use the history of infections and deaths to forecast. This involves inputting a numeric sequence of infected cases to a Recurrent Neural Network (RNN) to predict future infections or deaths. This strategy trains the RNN with supervised learning on the historic data. In addition to the numeric value of cases or deaths, these models will add dense embeddings to represent meta-information. This meta-information includes policy decisions such as the phase of lockdown in a given region.

These models can be useful for forecasting the amount of resources healthcare workers will need. According to Zeroual et al. [132] “accurate short-term forecasting of the number of new contaminated and recovered cases is crucial for optimizing the available resources and arresting or slowing down the progression of such diseases”. In their survey, Zeroual et al. [132] compare four different RNN architectures and a Variational Auto-Encoder (VAE) for spread forecasting. The VAE model has an encoder that compresses the history of observed cases into a latent vector which the decoder in turn maps into predicted cases in the future. Of particular note is the massive difference in how the time component is modeled in an RNN compared to a VAE. Their study predicts cases each day from January 22nd, 2020 to June 17th, 2020. We note that this only covers 148 days, or datapoints, for training the models. With this time-scale, they report the best performance with the VAE model. This is likely because the VAE has explicit mechanisms of sampling and variational inference that help regularize the latent space and avoid overfitting. We will discuss this issue further in Learning from Limited Labeled Data under Limitations of Deep Learning.

This approach of sequence modeling can be improved by adding more structure to the problem. This structure involves training sequence models for each county, state, country, or continent-level. These models can then be combined at later layers of the network such that the predictions of nearby regions can learned from one another. An important application of this modeling level is to decide how to resume travel. The SARS-CoV-2 pandemic has incurred a huge hit to travel and resulting economic activity.

**Fig. 17:**
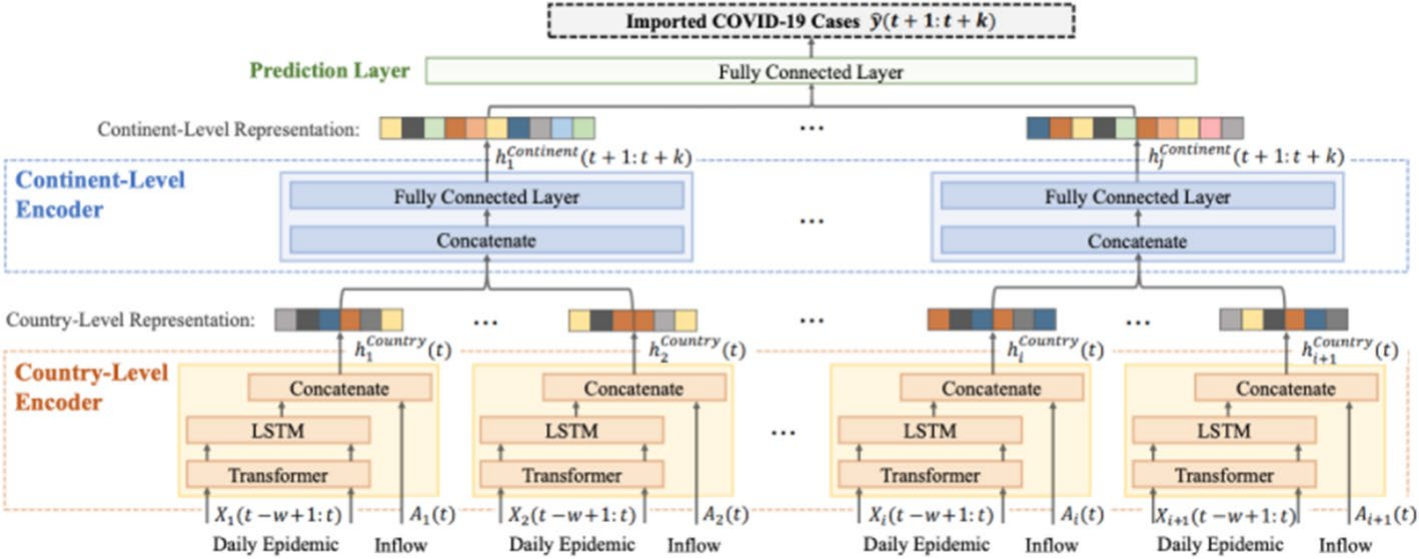
Hi-COVIDNet, using country-level and continent-level encoders to predict imported COVID-19 cases from travel (Image taken from Kim et al. [133])

Kim et al. [133] predict how much the virus would spread given travel from certain geographic regions. Their Deep Learning task architecture involves encoding travelers at the county-level and continent-level. At the county-level, the encoder is a combination of self-attention and recurrent layers to embed a history of daily infection numbers and the inflow of travelers from each country. The continent-level encoding aggregates the county-level encodings, passing them through an additional feed-forward layer. Their model design is pictured in Fig. 17. Readers may be interested in extending this work by exploring multi-task learning.

Le et al. [134] look at even more fine-grained, county- and state-level forecasting. Their dataset consists of confirmed cases, symptom surveys, movement range maps, community mobility, doctor visits, PCR tests per state, and weather. The objective is to disentangle region-specific factors such as demographics, enacted policies, and mobility from features of COVID itself. They propose a combination of RNNs and vector autoregressive models for this. This achieves very high-resolution models with respect to county and state-level forecasts.

#### SIR models

We have just described adding additional structure to the population in forecasting models. Under the scope of black-box forecasting, we compared the differences between sequence modeling with a single region of numeric cases or deaths compared to combining information from multiple regions. Now we will turn our attention to one of the core models of epidemiology, the SIR model. This model provides additional prior knowledge about what we know about infectious disease spread and the population. SIR models are more specific in modeling subsets of the population for accurate forecasting.

**Fig. 18:**
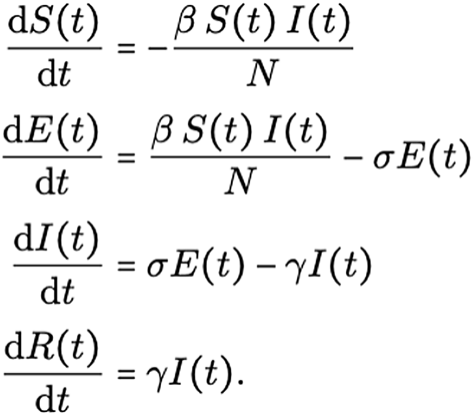
SEIR differential equations (Image taken from Dandekar and Barbastathis [135])

The SIR model is short for Susceptible (S), Infected (I), and Recovered (R). Sometimes these models are extended to include the Exposed (E) population as well, however this requires extremely detailed data. The model is a set of differential equations to compute the change in each S, I, E, and R population. These equations are shown in Fig. 18. The model makes a few important assumptions about the data. Firstly, there is a constant population, S + I + E + R = 1. Secondly, the rate of increase in infections is a function of contact and this contact occurs at a constant rate. Lastly, there is a constant rate of recovery.

The model answers questions given the initial Susceptible and Infected populations. These questions include, will the disease spread? What will be the peak number of Infected people? How many people will catch the disease? This initial population data allows us to solve for increase in infections as a function of the spread of the virus divided by the recovery rate. This is known as the R0, reproductive rate of the virus. If this reproductive rate is greater than 1, the number of infected is likely to increase.

**Fig. 19:**
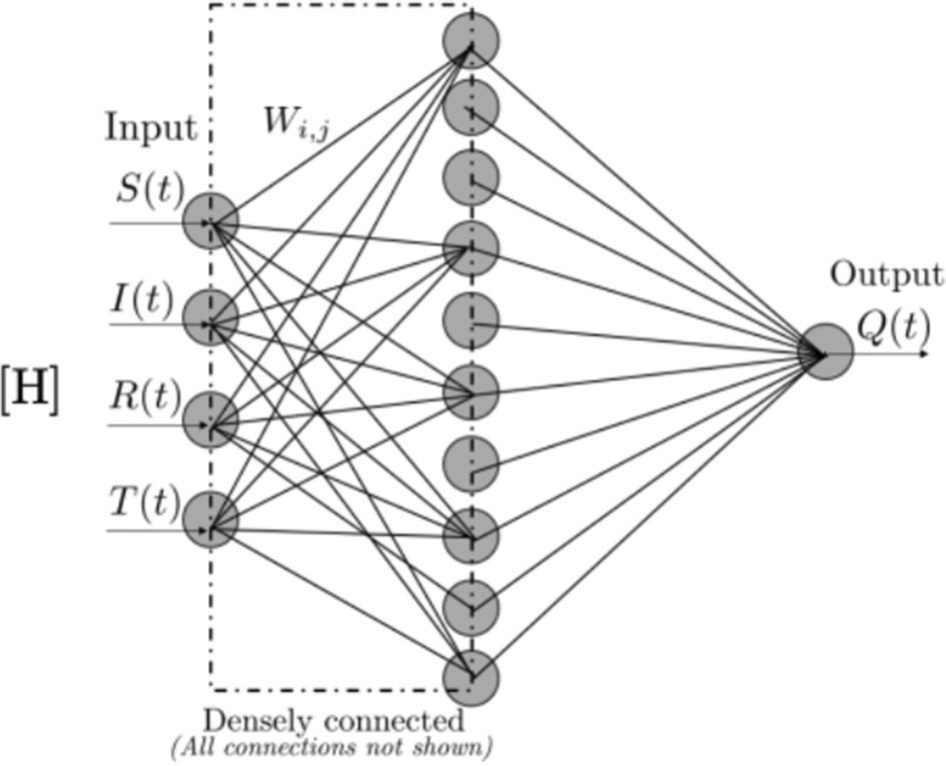
The Neural Network takes an input vector of the Susceptible, Infected, Recovered, and Quarantined Population (estimated from the previous time step) to model the quarantine strength (Image taken from Dandekar and Barabastathis [135])

The application of Deep Learning for SIR modeling is to reduce some of these simplifying assumptions. In many regions around the world, people have quarantined themselves to limit the spread. However, as described with black-box forecasting, there have been different phases of quarantine that vary over time. Dandekar and Barbastathis [135] introduce a 2-layer neural network to model the time-varying strength of quarantining. Figure 19 shows the input vector to the 2-layer neural network to predict the quarantine strength. The authors integrate this prediction into the differential modeling of the Infected population.

**Fig. 20:**
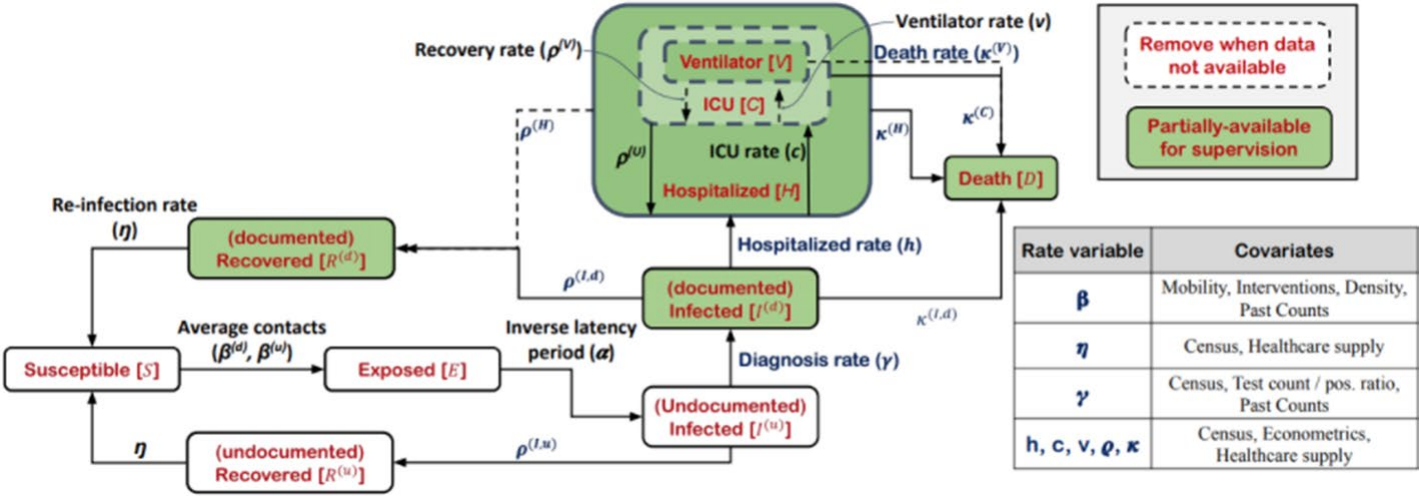
Further differentiation within populations in the SIR model (Image taken from Arik et al. [136])

In a similar way that SIR models add structure to improve black-box forecasting, researchers have further drilled into adding population structure. Arik et al. [136] extend the SIR Model by adding additional compartments. They further splitting the infected population into separate differential equations. The authors integrate undocumented infected and recovered cases, separate compartments for hospitalized, ICU and ventilator patients, re-infection rate. This more fine-grained population model is shown in Fig. 20. This introduces many more modeling parameters to be optimized with a Deep Neural Network.

#### Contact Tracing

Contact Tracing is another example of an application where we refer readers to our discussion of Data Privacy. The idea of Contact Tracing, more particularly Digital Contact Tracing, is to use cell phone interaction data through sensors to track contact between individuals. Users could also register for contact tracing by downloading a phone application. However, this would require everyone downloading the app to work effectively. This is especially important due to the phenomena of peak infectiousness preceding the onset of symptoms. Contact Tracing data would enable individuals to know if they have been exposed to someone carrying the virus, if they have visited a hotspot where the virus had been and might still be on surfaces, and thus quantify their risk of contracting it themselves. This quantification would improve over time as more data is collected about how these interactions actually lead to infections. This would also dramatically improve the infection rate parameter of the SIR models. There are no such public datasets that track individuals, however the Google Mobility Reports [78] do provide information about travel and contact between individuals at the city level.

**Fig. 21:**
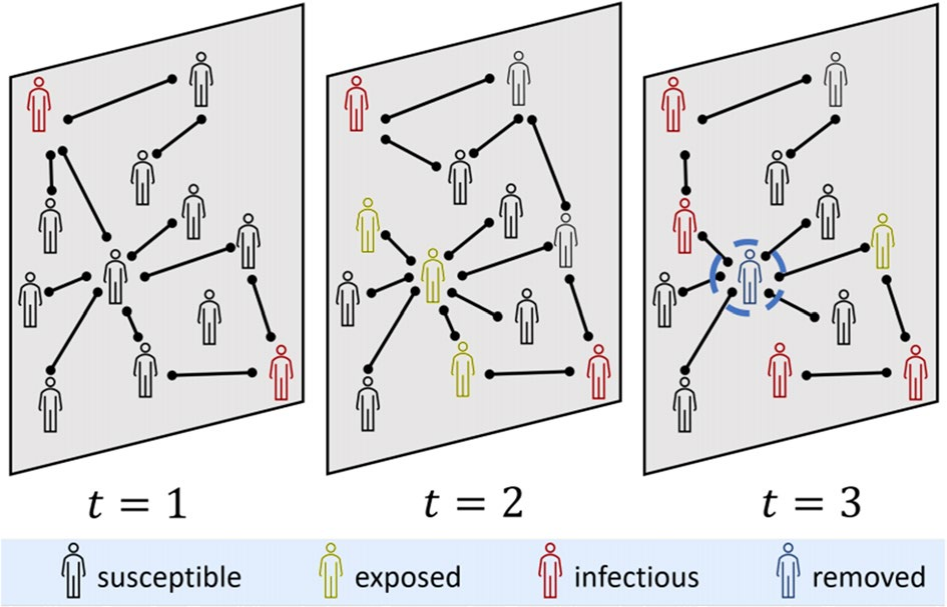
Graph-structured SIR models (Image taken from Meirom et al. [137])

Imagining we had this kind of data, Meirom et al. [137] present a promising strategy to control the spread using reinforcement learning and graph neural networks. Figure 21 highlights a way of viewing this graph in the lens of SEIR labels. Meirom et al. structure this as a sequential decision problem over a graph. Who should be tested? Who should quarantine themselves? These are the kind of decisions the model must make for individuals based on their contact. Meirom et al. use social network graph construction methods such as community-structured, preferential attachment, and statistics derived from real cellular tracking. These describe techniques for constructing an adjacency matrix with different features such as power-law or uniform connectivity. They simulate interactions and use graph neural networks to rank the candidates that should be prioritized for testing. In simulations this increases the number of healthy people by 25% and contains the epidemic 30% more often than supervised approaches. For real contact tracing datasets, these graph neural networks would become necessary to process a large population and large interaction data.

## Limitations of Deep Learning

Earlier, we discussed the current state of Deep Learning and how it fits into the broader context of Artificial Intelligence. In this section, we detail where Deep Learning falls short and how this can be problematic for the COVID-19 applications explored. In this section we explore Interpretability, Performance Evaluation, Learning from Limited Labeled Data, and Data Privacy. These limitations of Deep Learning each make up their own field of research. They raise important questions about practicality. Can we trust the predictions of Deep Learning models? Do we even have enough data and computing power to use Deep Learning?

The limitations of Deep Learning described in this survey are framed in the context of Human-AI Interaction. Topol [83] describes the absurdity of narrating Deep Learning applications in healthcare without this context, “this pitting of clinicians versus a machine is the antithesis of clinical practice, which invariably keeps humans in the loop”. This is most extremely illustrated in the case of automated diagnosis with life-or-death decision making. However, it is also extremely important to have painless user interfaces to Deep Learning models for Literature Mining, Misinformation Detection, or Drug Discovery.

We need new guidelines for user-interface design because when Deep Learning fails, it fails differently from traditional software. Amershi et al. [84] present detailed guidelines for Human-AI Interaction design based on these failures. They include 18 generally applicable design guidelines for deploying Deep Learning systems such as: make clear how well the system can do what it can do, make clear why the system did what it did, and learn from user behavior, to name a few. This section includes a presentation of Interpretability and Generalization Metrics. These are two of the most common failure modes for Deep Learning systems.

### Interpretability

Deep Learning achieves strong performance in the surveyed COVID-19 applications. However, it operates as a sort of black-box, and it is challenging to understand what caused it to make a certain prediction. This can be limiting to use Deep Learning for safety-critical applications. What will it take for a doctor to trust an automated diagnosis? Even outside of life and death situations, do we trust the model is correct? Will research scientists trust that BERT has correctly summarized the latest biomedical paper? Do we trust a Deep Learning model to identify the right protein target before spending millions of dollars developing a drug to attack it?

Our surveyed Deep Learning applications for COVID-19 require collaboration with humans. This branch of research is known as Human-AI interaction. Interpretability, also termed Explainability, is one of the key problems to solve in this field. Cambru highlights many benefits of explainability. These include justification of decisions to end-users, understanding and trusting the limitations of the system, providing insight for knowledge discovery, and diagnosing and improving Deep Learning systems.

Each of our surveyed applications in COVID-19 will benefit from these goals. Applications in Literature Mining can utilize explanations for the sake of knowledge discovery. As described previously, many of these Question Answering systems doubly implement Information Retrieval. This Information Retrieval reveals the most relevant documents that influenced the predicted answer. We view this as a similar motivation to providing explanations for knowledge discovery. However, we may further want to implement the tests we will describe next to probe for explanations in the retrieval system as well.

A user whose social media post has been flagged as “misinformation” may be irritated by the tagging and request justification. Sociologists relying on Public Sentiment Analysis systems would want to know words or phrases the model is most activated by. Medical Image Diagnosis is the surveyed application where we believe interpretability is the most important. Imagine a doctor is confident a patient has COVID based on reported symptoms, but the neural network classifies a non-COVID pneumonia. The doctor would like to have more than a logit score to judge this classification.

Under the scope of Ambient Intelligence applications, we imagined Computer Vision systems that perform tasks such as evaluating the performance of a surgeon. If the surgeon receives a poor mark, an explanation may be demanded in a similar vein as the irritated social media user with a flagged post. A Vision-based Robotics system tasked with sorting bottles may have been left unattended and performed a series of incorrect classifications. Here we need interpretability to help debug the system. Precision Diagnostics need interpretability similar to the emphasized importance of interpretability for Medical Image Diagnosis. We note that the multi-modal nature of Precision Diagnostics that combine genetic information with electronic health records and RT-PCR testing or medical images as well, could make this especially challenging.

We have a similar view of the role of interpretability in Protein Structure Prediction as Literature Mining for question answering. We imagine a nearest-neighbor style analysis would be very useful to understand why a certain structure was predicted. Drug repurposing candidates selected by querying knowledge graphs are very easily interpreted. However, once we start embedding nodes and edges and mining candidates with graph neural networks, interpretability is much more challenging. Interpretability with black-box or SIR-structured forecasting models may be one of the most important applications of these models altogether. We are not just interested in how many COVID-19 cases there will be in the next month, we want to know why the model has decided on this number.

Having motivated the application of explainability for Deep Learning applications, we describe cutting-edge approaches to interpretability. Cambru describes major research directions to achieve this. These are post-hoc explanatory methods and self-explanatory neural models that generate natural language explanations. Post-hoc explanatory methods refer to probing trained neural network models. We will describe looking at what part of the input caused a prediction, what activates certain neurons, and representation analysis.

The first question we explore is: what part of the input caused the network to make this decision? Clark et al. [138] explores what BERT looks at. This paper reports the relationship between the attention weights and their inputs at early layers of the network. This becomes more challenging to decode at deeper layers of the network. Tang et al. [139] explicitly limit the attention of a vision-based Reinforcement Learning agent, such that there is no ambiguity about where the model might be looking at.

Another question explored under the interpretability is, what activates certain neurons? Some researchers have looked at optimizing an image map to maximize the activations of an individual or set of neurons [140]. Yin et al. [141] generate images from activations for the sake of providing a knowledge distillation [142] set for compressing the network. Riberio et al. [143] propose the LIME framework to sample around a data point to provide analysis of what caused the prediction.

Another question we explore under interpretability is, what is captured in the representations? This area of research is also referred to as representation analysis. A common approach to this is to compress the high-dimensional representations into 2- or 3-dimensional space for visualization. Some common approaches to this are t-SNE [15] or UMAP [16]. These algorithms have recently been implemented in CUDA to run on GPUs and made accessible through libraries such as RAPIDS [144]. Chan et al. [145] describe using t-SNE-CUDA to embed datasets as large as ImageNet. Chan et al. note that “t-SNE-CUDA significantly outperforms existing methods with a 50–700x speedup without significantly impacting cluster quality” [145].

In WT5 [146], Narange et al. train a model to state why it made a certain prediction. The authors describe how the text input-text output unifying framework of NLP tasks facilitates this idea. Notably, the text-to-text task setup enables multi-task learning with a small labeled set of answers and explanations and a much larger set of answers only.

### Generalization metrics

Deep Learning models are typically evaluated by reporting performance metrics on a held-out test set. These performance metrics include accuracy, area under the true positive, false positive rate curve (AUC), and precision-recall, to name a few. Metrics other than accuracy are usually reported in instances of class imbalance, or to highlight performance on a particular class of interest. However, this performance reporting is insufficient for many of the surveyed applications. We are interested in how the model will generalize to distribution shift that may be encountered once the model has been deployed.

We define generalization as the performance difference on data sampled from a different distribution than the training set. When dividing data into train and test splits, the phenomena of overfitting has been a proxy for measuring generalization. We define overfitting as a test that says as long as the test error continues to decrease with the train error, generalization ability is intact. However, Nakkiran et al. illustrate the phenomena of “Deep Double Descent” where there is an empirically observed trend from overfitting to an interpolation threshold. With respect to model size or training duration, deep models appear to be overfitting, but then achieve generalization ability. We present double descent to highlight that overfitting, as we have defined it, is a poorly understood test for generalization in Deep Learning.

Our surveyed applications for COVID-19 need generalization ability for reliable deployment. We are not only interested in mining the existing literature on COVID-19, but we want to deploy these systems to adapt to new papers and provide new insights. Deployed Misinformation Detection models may need to generalize to new language used by spreaders as they try to game the system. Similarly, Public Sentiment Analysis models may need to adjust to new styles of using language. Generalization in Medical Image Analysis is extremely important due to the inherent variation in lung sizes and existing conditions. Ambient Intelligence systems need to be able to generalize to patients of all different heights, skin colors, or clothing. Our discussion of Vision-based Robotics is self-evident in the need for generalization. A disinfecting robot that can enter any room to locate and disinfect surfaces needs a massive generalization ability. Even robots used for sorting objects in manufacturing must be robust to rotations and lighting changes.

Our surveyed approaches to Precision Diagnostics rely heavily on generalization ability as well. This include generalizing to novel patient histories, genetic profiles, or RT-PCR testing results. Protein Structure Prediction models need to generalize in order to reliably predict the coronavirus shell proteins as soon as it was encountered. Drug Repurposing approaches need to generalize to explore new connections in the network. Forecasting models cannot be thrown off by anomalies in the data and need to react to novel configurations of quarantine protocol or neighboring regions’ infection spread. Having briefly motivated the need for generalization in these applications, we will further describe weaknesses of these models and directions for solutions.

An alarming moment for Deep Learning was the success of adversarial examples. Goodfellow et al. [147] showed that you could add an optimized noise map to a panda image such that it was classified as a gibbon with 99.3% confidence (Fig. 10). This image has become the poster for skeptics of Deep Learning. Within the scope of interpretability, we described how we could optimize an image to maximally activate certain neurons, such as DeepDream. This results in high-frequency, static-like features, similar to the noise map added to the panda image. Under the scope of generalization metrics, adversarial examples highlight that we need better tests for generalization. Within this issue, we define and explore two subsets of generalization metrics and human-constructed behavior tests.

As mentioned previously, most Deep and Machine Learning workflows take a dataset and randomly split it into training, validation, and test sets. Hyperparameters are fine-tuned using the validation set as a proxy and then test set performance is supposed to be a good proxy for generalization. However, real-world test distributions rarely overlap with this training set in this way. Measuring the distribution shift that occurs in a real training set is one of the most challenging areas of Deep Learning research. Winkens et al. [148] look at how contrastive learning can improve the ability to detect out-of-distribution inputs. At least with this functionality the model will output that an input is out-of-distribution, rather than a confident misclassification.

In addition to failure with respect to adversarially optimized noise maps, some models fail on simple, commonsense reasoning tasks. Ribeiro et al. [149] propose the CheckList evaluation system to test language models on linguistic capabilities such as negation and vocabulary. A solution to these behavior tests, and adversarial examples would be to simply train the model on this task data. Clark et al. [150] show that Transformers can chain facts together if they are explicitly trained to do so. This sounds like a great idea, but in practice a phenomenon referred to as catastrophic forgetting occurs. As neural networks optimize themselves to the latest batch of data and loss function, they “forget” previously learned examples.

We additionally highlight the problem of updating the knowledge contained in a neural network, particularly language models, when presented with new information. This is very important for our applications on Literature Mining and Misinformation. For example, correcting when an event occurred. We have discussed the representational difference between knowledge implicitly stored in the weights of a neural network and explicitly structured in knowledge graphs. When presented with a new fact, a knowledge graph can re-arrange edges and instantly integrate this new information. However, neural networks cannot be updated as easily. Stepping a language model through masked language modeling with the new fact will not completely integrate the knowledge into all of its predictions. A promising approach to this is the emphasis on evidence retrieval such as Retrieval-Augmented Generation [151].

### Learning from limited labeled datasets

The performance of Deep Learning improves with increasing amounts of data [152]. Even if the data is not labeled, such as how GPT-3 [4] learns language representations, it improves the success of Deep Learning. Further, these models improve dramatically with data that is more in-domain for the downstream task [23]. An additional million images from Instagram would not be as useful as 1000 lung CT scans for COVID-19 detection. Many areas of Deep Learning research cite that they are looking for the “ImageNet” moment in the given field. This references the success of a large labeled dataset to facilitate supervised representation learning. The current state of Deep Learning relies on these large datasets for improved performance. This is problematic for a pandemic response situation where quick response is crucial. Creating large datasets for many of our surveyed healthcare applications such as Medical Image Analysis or Precision Diagnostics is extremely challenging due to the data privacy issues we discuss in the next section. Nearly every COVID-19 application we surveyed would benefit from more labeled data.

Deep Learning problems usually have a small labeled dataset and a large unlabeled dataset. This is where we can turn to semi- and self-supervised learning. Self-supervised learning describes constructing a supervised learning task automatically from unlabeled data. For example, we can algorithmically rotate images and derive the rotation label from the pre-processing. Training the models on these kinds of tasks leads to useful representations that can be transferred to our supervised learning problem. Semi-supervised learning describes a similar idea, alternating between self-supervised learning on the unlabeled dataset and supervised learning with the labeled set. Of core importance here is that the unlabeled data is at least somewhat in-domain with the downstream task. For example, in COVID-19 diagnosis from radiographs, unlabeled chest radiographs are much more useful than ImageNet or landscape images.

In some cases, we can inject priors in the dataset that simultaneously increase the size of the dataset to prevent overfitting and inform the model which features it should be invariant to. This process is known as Data Augmentation. In the image domain, these priors refer to rotation or translational invariance. This is done by rotating the images algorithmically while preserving the label and applying the supervised loss on the augmented image. In text, this can be done by swapping out words with synonyms. This has mostly been explored in image data, with growing interest in text as well.

Another approach to learning from limited labeled data is meta-learning. We define “meta-learning” as learning from a few demonstrations, or leveraging information from k tasks to learn task k + 1. This is opposed to meta-learning to describe outer-inner loop optimization more generally and areas of research such as Neural Architecture Search. GPT-3 [4] demonstrates an impressive few-shot learning ability where the task is demonstrated in the input context window, such as “example 1: label 1; $\cdots$, test example: label ?”. Outer, inner-loop optimization for few-shot learning has advanced considerably with models such as MAML [153].

### Data privacy

Our previous section described that Deep Learning models performance better with larger datasets. In our coverage of Medical Image Analysis, we looked at Transfusion from Raghu et al. [83], which shows that out-of-domain data like ImageNet has little benefit for medical imaging tasks. The question is clear, how do we build large medical image datasets?

An issue with constructing these datasets is privacy. Imagining the role of Deep Learning in precision, tailor-made medicine and diagnostics, we would expect performance to improve by looking a massive collection of patients’ EHRs, genomes, blood testing results, family history, etc. However, most patients would not feel comfortable revealing such intimate data to a potentially hackable centralized database. The question as data scientists is, “can we answer questions using data we cannot see?” [85]. This introduces the first solution to privacy-preserving Deep Learning, Federated Learning.

**Fig. 22:**
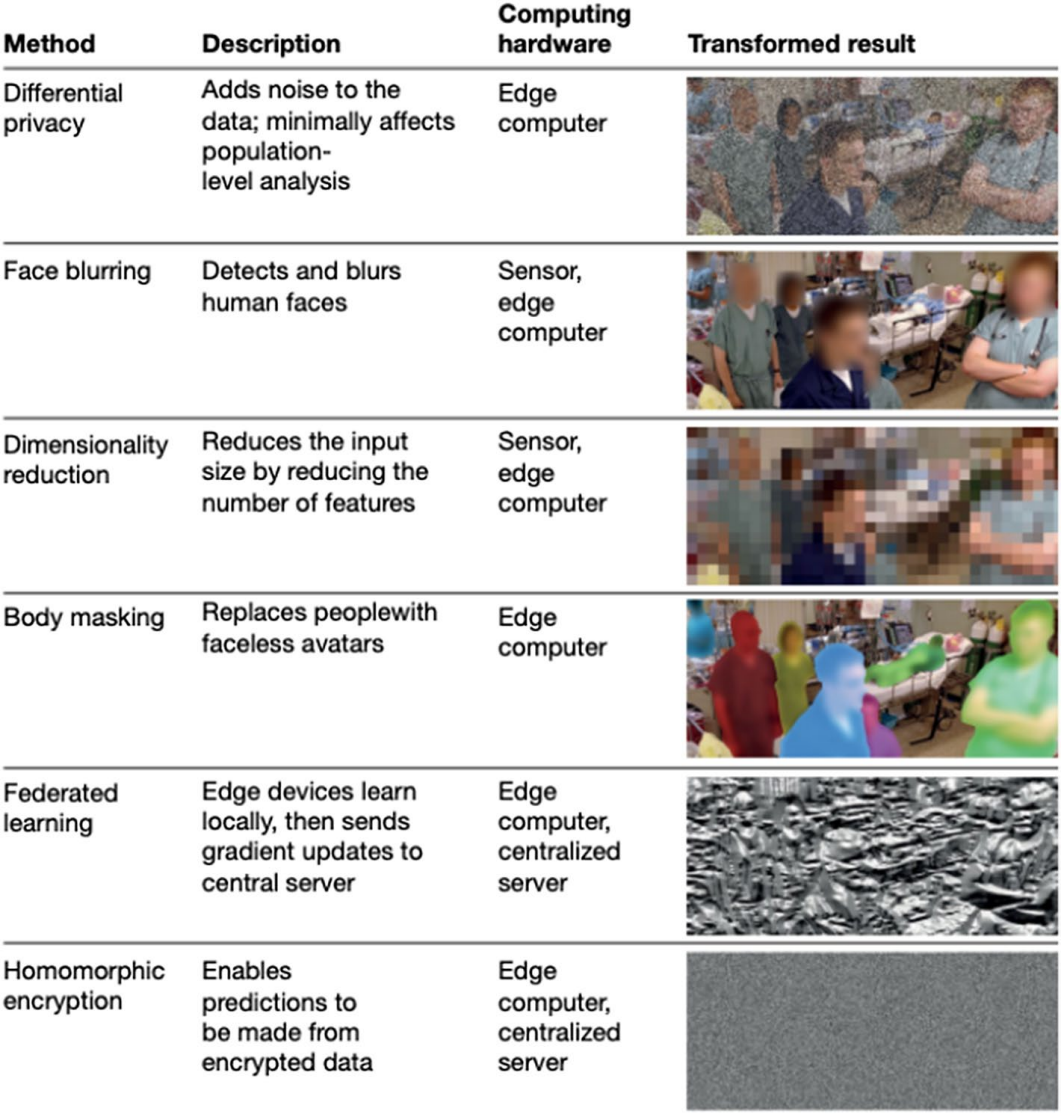
Privacy-preserving techniques explored in applications of Ambient Intelligence (Image taken from Haque et al. [10])

The core idea of Federated Learning is that there is no massive, centralized database. Copies of the model are sent to train on a locally stored database and then they are sent back to a centralized model weight database. This is a great first step, but some studies have shown that you can still recover the data from the model’s weights. Fredrikson et al. [86] use adversarial attacking to recover face images from a facial recognition given only black-box access to a person’s name and the confidence scores from the model. Stronger systems have been developed in response. These systems aim to guarantee a quantitative level of privacy through Differential Privacy. We refer interested readers to the privacy preserving Deep Learning framework developed by Ryffel et al. [87]. We additionally refer interested readers to Fig. 22 for an overview of privacy techniques used in Ambient Intelligence, developed by Haque et al. [10].

We note that data privacy is especially problematic when the patient can be identified from the data. This is not much of an issue with chest radiographs alone, but can be problematic with metadata associated with it, or stored in electronic health records. We additionally refer readers to a survey the use of recurrent neural networks for de-identification of Electronic Health Records (EHR) from Leevy et al. [154]. This survey describes the use of Deep Learning to identify and remove patient identification information from free text information.

## Discussion

The integration of Deep Learning with Biology and Healthcare is an exciting path forward in advancing technology. This survey highlights many ground-breaking applications to make this trend apparent. We encourage Deep Learning researchers to think about broad applications, and participate in the exercise of identifying problems in a given domain, such as COVID-19 or pandemic response. Composing this survey required taking an all-encompassing view of Deep Learning research. It is extremely difficult to acquire expertise across applications as diverse as Information Retrieval, Image Classification, or Protein Structure Prediction. However, we believe that the unifying reduction of data into input tensors, and tasks into learning variants, poses a common framework for many advancements in Deep Learning to disperse across applications. Improvements on the supervised learning process or on different limitations of Deep Learning from Data Privacy to better Generalization Metrics have a massive downstream result.

One of the goals of this survey was to help readers think about how their data is inputted to Deep Neural Networks and how they can construct a learning task. Data such as images are inputted as pixel grids, whereas categorical variables are embedded into dense representation tables [155]. With respect to learning, we have primarily focused on supervised, semi-supervised, and self-supervised learning. We have only briefly mentioned learning algorithms such as multi-task training or reinforcement learning. We encourage readers to explore these areas as well. In our literature review, we found that most Deep Learning research in COVID-19 focuses on novel combinations of learning tasks or on building new datasets and annotation protocols.

Given the current trend in Deep Learning research, emerging applications in Natural Language Processing are very compelling. NLP, powered by Deep Learning, is undergoing an evolution. GPT-3, a 175 billion parameter transformer language model, demonstrates a remarkable ability to complete fill-in-the-blank text prompts. This survey presents many downstream applications that benefit from the success of these language models. On the near horizon, we expect dramatic improvements in text classification, information retrieval, question answering, and summarization.

Our literature review has inspired further investigation in Literature Mining systems. Language Models are getting better and better at generating text that is coherent and factually accurate [37]. What would it require to ask GPT-3, “How is protein structure prediction formulated as a Deep Learning problem?” The application of this is very clear, although it may be a bit of a moonshot project. However, recent advances in neural information retrieval, efficient transformer design, new datasets, and self-supervised learning tasks hint that NLP-powered Literature Mining may just be scratching the surface.

These recent advances will also dramatically improve the ability to automate knowledge graph construction. This survey explored the construction of the CoV-KGE graph with 15 million edges collected across 24 million PubMed publications. Applying Deep Learning for graph representation learning at scale is still a relatively new and under-explored area of research. We expect the integration with biologists to seed Deep Learning systems with prior knowledge of virus-host interactomes and other kinds of interaction networks such as Protein-Protein Interactions (PPIs), to transform the capability of these algorithms. The discovery of new relations between entities as a link prediction problem on knowledge graph embeddings looks very promising.

The growth of Computer Vision will require an adjustment for society at large. Will patients trust an automated radiograph diagnosis? Will patients feel comfortable having their ICU activity monitored every minute of the day? Vision-powered robotics range on this scale. It is hard to imagine someone would object to a robotic disinfection sprayer, but robotic surgery is a more uncomfortable idea. This transformation will require further investment in safety and education.

Many papers report the success of Transfer Learning from models pre-trained on ImageNet. This is done despite findings from Raghu et al. [83] that challenge the benefit of this. This observation highlights the need for Literature Mining systems. How many of these researchers discovered Raghu et al.’s paper in their search for related works? Machine Learning researchers need search engines where they can send queries such as “What is the benefit of ImageNet Transfer Learning for Medical Image Analysis?”. We also note that this technique is likely so popular due to the ease of use. Deep Learning frameworks such as TensorFlow, Keras, and PyTorch provide very accessible interfaces to models with ImageNet trained weights. There is no need for the researchers to pre-train on ImageNet themselves.

The trend in Medical Image Analysis is to find new ways of annotating data or assembling tasks for representation learning. We surveyed interesting developments in image-text contrastive learning with text reports, human-in-the-loop annotation, and weak supervision. We are very excited about the image-text contrastive learning research explored by Zhang et al. [97]. Throughout this survey, we have mostly explored applications in a single domain. This describes only processing text or only processing image data. Vision-language representation learning at the same time is a promising step towards “grounded language learning”. We refer interested readers in this research direction to Brisk et al. [156]. Vision-language learning has recently improved GLUE benchmark performance [157]. ConVIRT [97] is an interesting direction to bring a language “grounding” to medical image processing.

Realizing the potential of Precision Diagnostics is one of the most important technological challenges of the 21st century. This begins with structured clinical data [158], and graduates to Deep Learning models processing structured and unstructured, multi-modal data. Integrating structured and unstructured data is challenging, but unstructured text data, such as clinical notes [159], can improve diagnosis. Blending multi-modal data, such as blood tests with CT scans, is another challenge suitable for Deep Learning. We note that there are not many public datasets of this type for development. One of the best open-source datasets is MIMIC-III [160], but even this dataset only contains about 60,000 records at the time of this publication. We again emphasize the importance of developing algorithms for Data Privacy that aids in developing these datasets.

Protein structure prediction has an interesting analogy with Information Retrieval problems. Similar amino acid sequences with experimentally verified structures are found through Multiple Sequence Alignment (MSA) and concatenated to the input representation for systems such as AlphaFold. MSA is a similarity algorithm based on dynamic programming which has a quadratic running time. Vector space similarity search can be dramatically sped up by computing centroids in the index. A representative centroid index can approach the constant running time of a hash function. As more amino acid sequences are verified and added to this database, currently containing 158 K instances, this search will become more important.

We also highlight the limitations of Deep Learning. Firstly, the current generation of Deep Learning systems must be designed with Human-AI interaction in mind. We do not expect these systems to operate entire autonomously. These models should have some degree of interpretability, some sense of expected out-of-distribution examples, and they should be able to learn without millions of labeled examples. Labeled or not, these models need data. The data that helps solve truly consequential problems such as Precision Diagnostics requires careful consideration of Data Privacy.

## Conclusion

In conclusion, we have presented many applications of Deep Learning to fight COVID-19. SARS-CoV-2 and COVID-19 have brought about many new problems for humanity to solve. Our survey provides a description of how some of these problems can be solved with Deep Learning. We have described how different data types are inputted to Deep Neural Networks and how tasks are constructed as learning problems. These applications are explored across data domains in Natural Language Processing, Computer Vision, Life Sciences, and Epidemiology.

We have also covered some of the most pressing limitations of Deep Learning. This includes challenges of Interpretability, Performance Metrics, Learning from Limited Labeled Data, and Data Privacy. We have covered some potential solutions to these limitations as well. We are optimistic in the transformative potential of these applications and believe their core limitations can be overcome. We hope this introduction will help readers narrow their interest and pursue these applications.

## Data Availability

Not applicable.
